# Molecular and Biochemical Insights Into Early Responses of Hemp to Cd and Zn Exposure and the Potential Effect of Si on Stress Response

**DOI:** 10.3389/fpls.2021.711853

**Published:** 2021-09-03

**Authors:** Marie Luyckx, Jean-François Hausman, Kjell Sergeant, Gea Guerriero, Stanley Lutts

**Affiliations:** ^1^Groupe de Recherche en Physiologie végétale, Earth and Life Institute – Agronomy (ELI-A), Université catholique de Louvain, Louvain-la-Neuve, Belgium; ^2^Environmental Research and Innovation Department, Luxembourg Institute of Science and Technology, Esch-sur-Alzette, Luxembourg

**Keywords:** cadmium, zinc, hemp, phytoremediation, heavy metal

## Abstract

With the intensification of human activities, plants are more frequently exposed to heavy metals (HM). Zinc (Zn) and cadmium (Cd) are frequently and simultaneously found in contaminated soils, including agronomic soils contaminated by the atmospheric fallout near smelters. The fiber crop *Cannabis sativa* L. is a suitable alternative to food crops for crop cultivation on these soils. In this study, Cd (20 μM) and Zn (100 μM) were shown to induce comparable growth inhibition in *C. sativa*. To devise agricultural strategies aimed at improving crop yield, the effect of silicon (Si; 2 mM) on the stress tolerance of plants was considered. Targeted gene expression and proteomic analysis were performed on leaves and roots after 1 week of treatment. Both Cd- and Zn-stimulated genes involved in proline biosynthesis [pyrroline-5-carboxylate reductase (*P5CR*)] and phenylpropanoid pathway [phenylalanine ammonia-lyase (*PAL*)] but Cd also specifically increased the expression of *PCS1-1* involved in phytochelatin (PC) synthesis. Si exposure influences the expression of numerous genes in a contrasting way in Cd- and Zn-exposed plants. At the leaf level, the accumulation of 122 proteins was affected by Cd, whereas 47 proteins were affected by Zn: only 16 proteins were affected by both Cd and Zn. The number of proteins affected due to Si exposure (27) alone was by far lower, and 12 were not modified by heavy metal treatment while no common protein seemed to be modified by both CdSi and ZnSi treatment. It is concluded that Cd and Zn had a clear different impact on plant metabolism and that Si confers a specific physiological status to stressed plants, with quite distinct impacts on hemp proteome depending on the considered heavy metal.

## Introduction

Plants often experience various biotic and abiotic stresses during their life cycle. The abiotic stresses include mainly drought, salt, temperature (low/high), flooding, and element deficiency/excess (Ahmad P. et al., 2016). Among the naturally occurring elements, 53 are classified as heavy metals (HM), the majority of HM does not play an essential role in plants although some HM such as zinc (Zn) and copper (Cu) are the essential elements for eukaryotic cells (Bhat et al., 2019). With the intensification of human activities, such as mining and industrial activities and the excessive utilization of poor quality phosphate fertilizer, plants are more and more exposed to HM, which hamper crop growth and yield to a great extent (Ahmad P. et al., 2016; Jian et al., 2020). HM-induced stress represents, therefore, a critical challenge for agricultural productivity (Ghosh et al., 2017; Kosová et al., 2018).

In numerous areas of the world, HM-polluted agricultural soils cannot be safely used anymore for edible crop production due to risks for human health, and only nonedible plant production remains possible (Feng et al., 2020). HM affect crop growth and yield through a negative impact on photosynthesis and also affect root growth, which alters the water balance and nutrient assimilation, thereby affecting their translocation to the abovementioned ground plant parts and biomass production (Singh et al., 2016). In the molecular levels, HM exposure can affect protein synthesis and structure, block the functional groups of metabolically important molecules, supersede the functionality of essential metals in biomolecules, affect the integrity of membranes, and increase the generation of reactive oxygen species (ROS) (reviewed by Emamverdian et al., 2015; Singh et al., 2016).

The initial step of plant cell behavior toward any environmental constraint is stress sensing (Hossain and Komatsu, 2013): the changes in ambient conditions are sensed by the receptors in the plasma membrane, inducing signaling pathways to transfer a stress signal from the plasma membrane to nucleus and leading to the changes in gene expression (Singh et al., 2016; Kosová et al., 2018). The sequestration of HM into the cell wall (CW) is probably the first strategy to limit the entry of HM into plant cells (Ye et al., 2012; Fernández et al., 2014; Parrotta et al., 2015; Nawaz et al., 2019). Pectins can adsorb HM via their carboxylic groups, and the lignification of the CW could be a strategy to limit HM entry into the cell by making the CW less permeable (Pejic et al., 2009; Vukcevic et al., 2014; Gutsch et al., 2019). Once inside cells, the regulation of a plasmodesmata aperture through callose synthesis and deposition may limit the transfer of metal ions from one cell to another (Singh et al., 2016; O’Lexy et al., 2018). To limit their toxicity, HM has to be delivered to the appropriate subcellular compartment (Singh et al., 2016; O’Lexy et al., 2018). This can be achieved through ion chelation [phytochelatin (PC) and metallothionein (MT)]: the complex formed between a metal ion and a chelating agent is transported into the vacuoles where metal ions can no longer affect the functioning of the cells (Cobbett, 2000; Fernández et al., 2014; Jost and Jost-Tse, 2018). Using these mechanisms, many plant species have the potential to grow on contaminated sites, and some species can accumulate high concentrations of HM in their tissues.

*Cannabis sativa* L. (hemp) is a multipurpose crop, which is considered as a potential crop for cleaning the soil from HM due to its high biomass production, its long root system, and its capability to absorb and accumulate HM (Ahmad R. et al., 2016; Kumar et al., 2017). It is a promising species for fiber production on low HM-contaminated substrates (Hussain A. et al., 2019; Hussain R. et al., 2019; Pietrini et al., 2019). Both woody fibers (shivs) and cellulosic bast fibers are produced in the stem, and this organ, therefore, receives considerable attention to decipher the major molecular cues controlling the biogenesis of these two fibers (Guerriero et al., 2017a,b; Behr, 2018; Luyckx et al., 2021b). Luyckx et al. (2021b) reported that cadmium (Cd) and high concentrations of Zn reduced the diameter of primary bast fibers and that Cd negatively affected cellulose and lignin biosynthesis when high concentrations of Zn had an opposite effect. According to this study, only a minor proportion of proteins was affected by both Cd and Zn. Cd increased the abundance of enzymes from the tricarboxylic acid (TCA) cycle and negatively affected the proteins involved in CW deposition, whereas Zn had an opposite effect. According to Pietrini et al. (2019), *C. sativa* can grow on the soil containing 150 mg kg^–1^ Zn, whereas other studies revealed that this species is still able to cope, to some extent, with 500 mg kg^–1^ Zn (Angelova et al., 2004; Meers et al., 2005). Considering the mean values of Zn bioavailability in polluted soils (Kos and Leštan, 2004), it may be considered that the doses ranging from 50 to 150 μM Zn in nutrient solution correspond to realistic moderate stress for *C. sativa* (Zlobin, 2021).

To devise agricultural strategies aimed at improving crop yield, the effect of silicon (Si) on the stress tolerance of plants should be considered as a promising strategy (Luyckx et al., 2017). This element is not considered essential for plant growth and development, but the beneficial effects of Si fertilizers on plant growth and crop yields are now well documented in the literature (Keeping and Reynolds, 2009; Bhat et al., 2019). The commonly considered mechanisms contributing to Si-induced stress tolerance, including toxic metal immobilization in the soil, the stimulation of antioxidants, the coprecipitation of metals within plant tissues, the chelation of metal ions, compartmentation, the structural alterations of plant tissues, and the biochemical response triggering metabolic changes (Luyckx et al., 2017; Bhat et al., 2019). However, the precise molecular parameters involved in Si-induced adaptative processes have not been identified (Luyckx et al., 2017).

In addition to stems, which constitute the site of the differentiation of fibers, the behaviors of roots and leaves are of paramount importance for the whole survival of stressed plants. Roots are indeed the first organ to have direct contact with HM, regulate pollutant absorption, and transfer them to the shoot parts. Leaves provide energy for plant growth and control ion translocation through the regulation of transpiration. Lefèvre et al. (2014) demonstrated that Cd and Zn may exhibit a distinct distribution in leaf tissues and bind to the different ligands in *Zygophyllum fabago* (Syrian bean-caper). Leaf proteomics evidenced the protection of photosynthetically active tissues and the maintenance of cell turgor through the synthesis of proteins involved in the photosynthetic apparatus, C-metabolism, and the synthesis of osmoprotectants. As far as hemp is concerned, Luyckx et al. (2021a) recently demonstrated that Cd affects photosynthesis through non-stomatal effects and increased glutathione (GSH) and PC synthesis in the roots while exogenous Si decreased Cd accumulation in all organs and improved water use efficiency. Hemp leaves accumulate Si in the form of silica precipitating in epidermal cells and the basal cells and shafts of non-secreting trichomes (Guerriero et al., 2019; Berni et al., 2020), but the molecular link between Si accumulation and physiological protection remains unknown.

The aims of the present study are as follows: (1) to determine the impact of Cd and Zn on the expression of key genes in the roots and leaves of *C. sativa*, (2) to assess the effect of these conditions on the leaf proteome, and (3) to analyze the impact of Si on those molecular responses in stressed and unstressed plants.

## Materials and Methods

### Plant Materials and Growth Conditions

The seeds of a monoecious hemp fiber variety (*C. sativa* cv. Santhica 27) were sown in a loam substrate in greenhouse conditions. After 1 week, the obtained seedlings were transferred to the nutrient Hoagland solution [in mM: 2.0 KNO_3_, 1.7 Ca(NO_3_)_2_, 1.0 KH_2_PO_4_, 0.5 NH_4_NO_3_, and 0.5 MgSO_4_ and in μM: 17.8 Na_2_SO_4_, 11.3 H_3_BO_3_, 1.6 MnSO_4_, 1 ZnSO_4_, 0.3 CuSO_4_, 0.03 (NH_4_)_6_Mo_7_O_24_, and 14.5 Fe-EDDHA] in 25 L tanks: for each tank, 10 seedlings were adapted to plugged holes in a polystyrene plate floating at the top of the solution. Tanks (24) were placed in a phytotron under fully controlled environmental conditions (constant temperature of 24°C ± 1°C with a mean light intensity of 230 μmoles m^–2^s^–1^ provided by Phillips lamps (Philips Lighting S.A., Brussels, Belgium) (HPI-T 400 W), with a photoperiod of 16 h under a relative humidity of 65%). Half of the tanks received 2 mM Si in the form of metasilicic acid (H_2_SiO_3_) obtained from a pentahydrate sodium metasilicate (Na_2_SiO_3_⋅5 H_2_O), which was passed through an H^+^ ion exchanger resin IR 20 Amberlite type according to Dufey et al. (2014). Tanks were randomly arranged in the phytotron, and the nutrient solution was permanently aerated by the SuperFish Air Flow four pump. After 2 weeks of acclimatization, HM was then applied in the form of CdCl*2* (final concentration of 20 μM) and ZnCl*2* (100 μM). The pH of the solution was maintained at 5.5. The solubility of the added HM was confirmed by the Visual MINTEQ09 software. Six treatments were thus defined, considering the presence of HM and the concomitant presence or absence of Si, i.e., C (control: no HM and no Si), CSi, Cd, CdSi, Zn, and ZnSi (four tanks per treatment). Plants were harvested after 1 week of treatment. Plants from the same tank were pooled: hence, four pools containing six plants each were obtained for each treatment. Roots, leaves, and stems were separated and weighed. Some samples were incubated in an oven (70°C) for the analysis of iron content, whereas the remaining samples were frozen in liquid nitrogen and then stored at −80°C until subsequent biochemical, gene expression analysis, and proteomics.

### Ion Content, Glutathione, PC, and Proline Concentration

For Cd and Zn measurements, c.a. 50 mg dry matter was digested in 68% HNO_3_ and evaporated at 80°C. Minerals were incubated in HCl 37%-HNO_3_ 68% (3:1) until evaporation and dissolved in distilled water; ions were quantified by Inductively Coupled Plasma atomic emission spectroscopy (ICP) (Varian, type MPX, Palo Alto, CA, United States). Si was separately quantified after calcination as detailed by Luyckx et al. (2021b).

Glutathione [GSH and glutathione disulfide (GSSG)] was determined by high-performance liquid chromatography (HPLC) on frozen samples after derivatization by orthophthalaldehyde according to Cereser et al. (2001). Total nonprotein thiol (NPT) concentration was determined using Ellman’s reagent according to De Vos et al. (1992). PC content was evaluated as the difference between NPT and GSH levels. Proline was spectrophotometrically quantified at 520 nm using the acid ninhydrin method (Bates et al., 1973).

### Targeted Gene Expression Analysis

Total RNA was extracted from leaves and roots according to Guerriero et al. (2017a) and Mangeot-Peter et al. (2016) using the RNeasy Plant Mini Kit (Qiagen, Hilden, Germany) treated on-column with DNase I. The RNA concentration and quality for each sample were measured by using a Nanodrop ND-1000 (Thermo Scientific, Waltham, MA, United States) and a 2100 Bioanalyzer (Agilent Life Sciences, Santa Clara, CA, United States), respectively. The RNA integrity number (RIN) of all samples was higher than seven, and the ratios A260/280 and A260/230 were between 1.7 and 2.4. The extracted RNA was retrotranscribed into complementary DNA (cDNA) using the SuperScript II reverse transcriptase (Invitrogen, Waltham, MA, United States) and random primers, according to the instructions of the manufacturer. The synthesized cDNA was diluted to 2 ng/μl and used for the quantitative reverse transcription Polymerase Chain Reaction (PCR) (RT-qPCR) analysis in 384-well plates. An automated liquid handling robot (epMotion 5073, Eppendorf, Hamburg, Germany) was used to prepare 384 well plates. To check the specificity of the amplified products, a melt curve analysis was performed. The relative gene expression was calculated with qBase^PLUS^ (version 2.5, Biogazelle, Gent, Belgium) by using the reference genes (*eTIF4E, TIP41, F-box*, and RAN) (Mangeot-Peter et al., 2016). Statistics (ANOVA2) was performed using R (version 3.3.1).

The target genes belong to the candidates involved in photosynthesis [RuBisCO activase (*RCA*), RuBisCO (*RBCS*), and chlorophyllases (*CLH*)], aquaporin-mediating Si passage (*Lsi*), *NIP2-1* (Si channel), and *NIP2-2*, HM transport and sequestration [*MT2B* and phytochelatin synthase (*PCS2*)], signaling [ethylene-responsive factor (*ERF1*) and gibberellin receptor (*Gibbrec*)], proline biosynthesis [pyrroline-5-carboxylate reductase (*P5CR*) and pyrroline-5-carboxylate synthetase (*P5CS*)], stress response [iron superoxide dismutase (*FSD*), ascorbate peroxidase (*APX*), and heat shock protein (*HSP*)], and phenylpropanoid pathway [cinnamyl alcohol dehydrogenase (*CAD*) and phenylalanine ammonia-lyase (*PAL*)]. Those genes were selected based on previous studies (Guerriero et al., 2017a,b, 2019; Berni et al., 2020; Luyckx et al., 2021b) and were analyzed in the appropriate organ according to these works.

The corresponding primers were designed using Primer3 Plus (http://www.bioinformatics.nl/cgi-bin/primer3plus/primer 3plus.cgi/) and verified with the OligoAnalyzer 3.1 tool (Integrated DNA Technologies, Coralville, IA, United States, http://eu.idtdna.com/calc/analyzer). Primer efficiencies were checked via quantitative PCR (qPCR) using six serial dilutions of cDNA (10, 2, 0.4, 0.08, 0.016, and 0.0032 ng/μl). For each considered gene, the selected primers and target organs are listed in the Supplementary Table.

### Proteomic Analysis

For each sample, 500 mg fresh matter of *C. sativa* leaves were homogenized in a Potter (Wheaton, IL, United States) homogenizer in 2 ml of homogenization buffer [50 mM Tris, pH 7.5 (HCl), 2 mM EDTA, 5 mM dithiothreitol (DTT)], protease inhibitor mix [1 mM phenylmethylsulfonyl fluoride (PMSF), 2 μg/ml each of leupeptin, aprotinin, antipain, pepstatin, and chymostatin, 0.6% w/v polyvinylpolypyrrolidone, 30 mM spermine]. The homogenate was centrifuged for 5 min at 2,000 rpm and 4°C. Protein extracts were centrifuged at 2°C for 30 min at 54,000 rpm (TLA55, Optima-Beckman-Coulter) to obtain a pellet of crude membranes and supernatant.

About 20 μg of each sample was transferred to 0.5 ml polypropylene protein LoBind Eppendorf Tubes and precipitated with the chloroform-methanol method (Wessel and Flügge, 1984); 20 μl of 100 mM triethylammonium bicarbonate (TEAB) was then added to reach pH 8.5. Proteins were reduced by 5 mM DTT and alkylated by 15 mM iodoacetamide. Proteolysis was performed with 0.5 μg of trypsin and allowed to continue overnight at 37°C. Each sample was dried under vacuum with a Savant Speed Vac Concentrator (Thermo Scientific, Hudson, MA, United States).

Before peptide separation, the samples were dissolved in 20 μl of 0.1% (v/v) formic acid and 2% (v/v) acetonitrile (ACN). The peptide mixture was separated by reverse-phase chromatography on a NanoACQUITY UPLC MClass system (Waters) working with the MassLynx V4.1 (Waters) software; 200 ng of digested proteins were injected on a trap C18, 100 Å 5 μm, 180 × 20 mm column (Waters) and desalted using isocratic conditions at a flow rate of 15 μl/min using a 99% formic acid and 1% (v/v) ACN buffer for 3 min. The peptide mixture was subjected to reverse phase chromatography on a C18, 100 Å 1.8 mm, 75 μm × 150 mm column (Waters) PepMap for 120 min at 35°C at a flow rate of 300 nl/min using a two-part linear gradient from 1% (v/v) ACN, 0.1% formic acid to 35% (v/v) ACN, 0.1% formic acid and from 35% (v/v) ACN, 0.1% formic acid to 85% (v/v) ACN, 0.1% formic acid. The column was re-equilibrated at initial conditions after washing 10 min at 85% (v/v) ACN, 0.1% formic acid at a flow rate of 300 nl/min. For online liquid chromatography-mass spectrometry (LC-MS) analysis, the nanoUPLC was coupled to the mass spectrometer through a nanoelectrospray ionization (nanoESI) source emitter.

Ion mobility separation-high definition mass spectrometry enhanced (IMS-HDMSE) analysis was performed on an SYNAPT G2-Si high definition mass spectrometer (Waters) equipped with a NanoLockSpray dual electrospray ion source (Waters). Precut fused silica PicoTipR Emitters for nanoelectrospray, outer diameters: 360 μm; inner diameter: 20 μm; 10 μm tip; and 2.5″ length (Waters) were used for samples, and Precut fused silica TicoTipR Emitters for nanoelectrospray, outer diameters: 360 μm; inner diameter: 20 mm; and 2.5″ length (Waters) were used for the lock mass solution. The eluent was sprayed at a spray voltage of 2.4 kV with a sampling cone voltage of 25 V and a source offset of 30 V. The source temperature was set to 80°C. The HDMSE method in resolution mode was used to collect the 15 min data after a 106 min injection. This method acquires MSE in positive and resolution modes over the m/z range from 50 to 2,000 with a scan time of 1 s with a collision energy ramp starting from ion mobility bin 20 (20 eV) to 110 (45 eV). The collision energy in the transfer cell for low-energy MS mode was set to 4 eV. For the post-acquisition lock mass correction of the data in the MS method, the doubly charged monoisotopic ion of [Glu1]-fibrinopeptide B was used at 100 fmol/ml using the reference sprayer of the nanoESI source with a frequency of 30 s at 0.5 ml/min into the mass spectrometer.

High definition mass spectrometry enhanced data were processed with the Progenesis QI (Nonlinear DYNAMICS, Waters) software using *C. sativa* NCBI database downloaded on October 8, 2019. The selection of carbamidomethylation as the fixed cysteine modification, oxidation as the variable methionine modification, and trypsin as the digestion enzyme was done, and one miscleavage was allowed.

### Microscopic Determination of Lignification and Cd Deposition in Leaves and Roots

Pieces of leaf tissue were rapidly excised from fresh leaves (no. 2; acropetal numbering) with a scalpel and dipped into tissue freezing media (O.C.T., Tissue Tek, Jung, etc.), and into propane cooled by liquid nitrogen. The plant pieces were next sectioned at 60 μm thickness using a Leica CM3050 cryotome (Leica, Wetzlar, Germany), placed in Al holders, and transferred to an Alpha 2-4 Christ freeze dryer (−50°C, 0.04 mbar, 3 days). Freeze-dried cross-sections were photographed using a digital camera (AxioCam) mounted on a Zeiss Axioscope two fluorescence microscope (wavelength: 405 nm). Cross-sections were also studied using X-ray fluorescence at beamline ID21 (ESRF).

### Statistical Analysis

Except for proteomic, four independent biological replicates and three technical replicates were analyzed for each condition. The normality of the data was verified using Shapiro–Wilk tests, and the data were transformed when required. The homogeneity of the data was verified using Levene’s test. ANOVA 2 was performed at a significant level of value of *p* < 0.05 using R (version 3.3.1) considering the type of HM treatment and the Si application as main factors. Means were compared using Tukey’s honestly significant difference (HSD) all-pairwise comparisons at 5% level as a *post hoc* test.

Proteomic analysis was performed two times, and it provided similar trends. The non-conflicting method was used as the relative quantification method. To identify statistically significant, differentially expressed proteins, the combined criteria of a minimum of three or more unique peptides, a 1.5-fold change ratio or greater, and the value of *p* < 0.05 in the Student’s *t*-test were adopted.

## Results

### Heavy Metal Impacts on Plant Growth

Cadmium decreased the dry weight of roots, stems, and leaves by 53, 62, and 71%, respectively, whereas Zn decreased the dry weight of roots, stems, and leaves by 34, 42, and 29%, respectively. Si mitigated the growth inhibition induced by Zn (+12% in roots and + 13% in shoots) and Cd (+21% in roots and +18% in shoots) in comparison to the exposure of plants to HM in the absence of Si (see details in Supplementary Figure 1).

### Ion Accumulation, GSH, PC, and Proline Content

**FIGURE 1:**
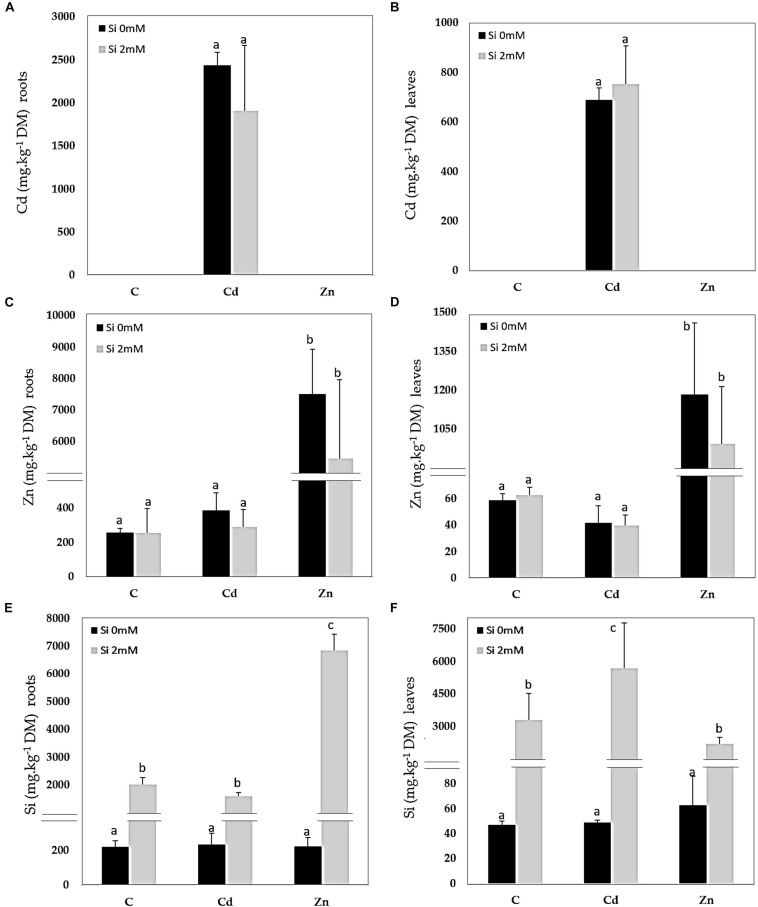
Cadmium (Cd), zinc (Zn), and silicon (Si) in roots **(A,C,E)** and leaves **(B,D,F)** of *Cannabis sativa* (cv. Santhica 27). Plants were exposed for 1 week to Cd (20 μM) or Zn (100 μM) in the presence or absence of Si (2 mM) [C: control plants not exposed to heavy metals (HM)]. The different letters indicate that the values are significantly different from each other [*p* < 0.05; Tukey’s honestly significant difference (HSD) all-pairwise comparisons].

**FIGURE 2:**
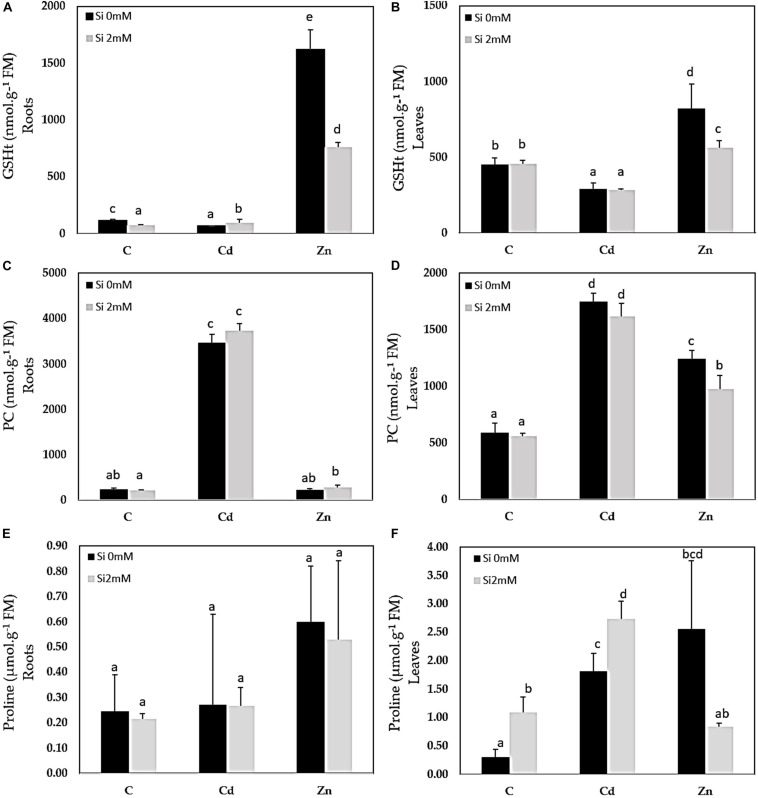
Glutathione (GSHt), phytochelatin (PC), and proline contents in roots **(A,C,E)** and leaves **(B,D,F)** of *C. sativa* (cv. Santhica 27). Plants were exposed for 1 week to Cd (20 μM) or Zn (100 μM) in the presence or absence of Si (2 mM) (C: control plants not exposed to HM). The different letters indicate that the values are significantly different from each other (*p* < 0.05; Tukey’s HSD all-pairwise comparisons).

Cadmium was detected in Cd-treated plants only and accumulated to higher concentrations in the roots than in the leaves (Figures 1A,B). Si had no significant impact on Cd accumulation. In Zn-treated plants, Zn also accumulated to a higher extent in the roots (Figure 1C) than in the leaves (Figure 1D): Si tended to reduce Zn accumulation in both organs although the recorded decrease was not significant. A similar root Si accumulation was recorded for control and Cd-treated plants (Figure 1E) while the Si accumulation in roots was the highest for Zn-treated plants. As far as the leaves are concerned (Figure 1F), the highest Si accumulation was recorded for Cd-treated plants. GSH accumulated in the roots of Zn-treated plants and additional Si strongly reduced such types of accumulation (Figure 2A). In the leaves, Cd decreased GSH content in the absence as well as the presence of Si (Figure 2B), whereas GSH content increased in response to Zn and to a higher extent in case of the absence compared to the presence of Si. PC accumulated mainly in the roots of Cd-treated plants (Figure 2C), and Si had no impact on this parameter. PC concentration was lower in the leaves than in the roots: the highest leaf PC concentration was recorded in Cd-treated plants (Figure 2D) and the lowest in control ones. Treatments had no significant impact on root proline content (Figure 2E). In the leaves, however, Si increased proline concentration in control plants (Figure 2F). Both Cd and Zn increased leaf proline concentration but Si had contrasting impacts as it increased leaf proline concentration in Cd-treated plants, but drastically reduced it in Zn-exposed ones.

### Gene Expression in Leaves and Roots

**FIGURE 3:**
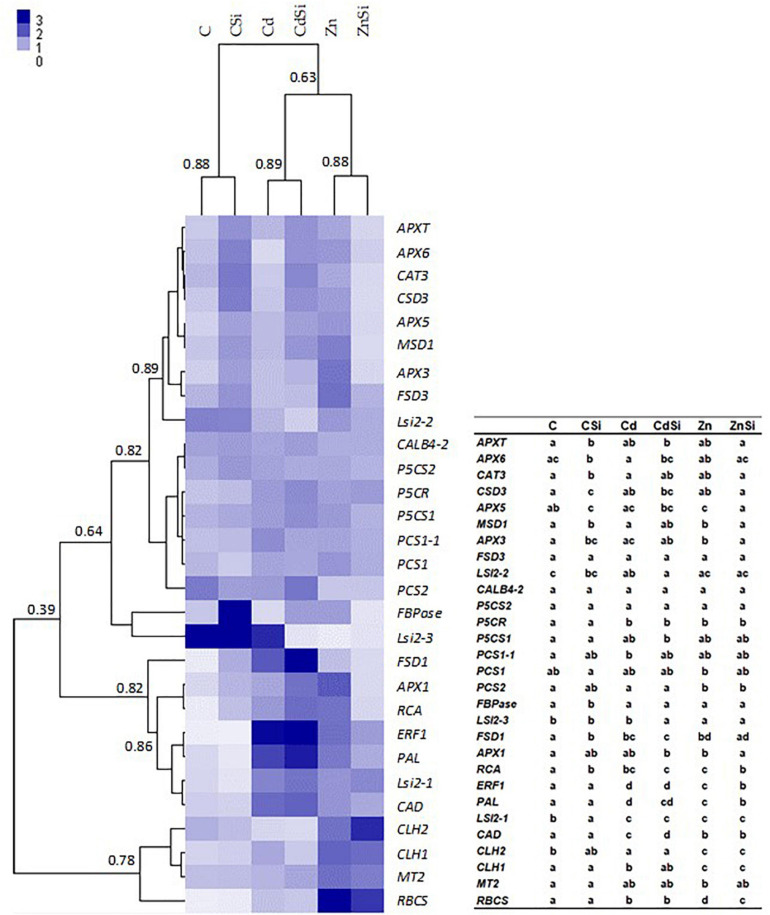
Heatmap hierarchical clustering showing the expression of genes assessed by quantitative reverse transcription PCR (RT-qPCR) in the leaves of hemp plants. Plants were exposed for 1 week to Cd (20 μM) or Zn (100 μM) in the presence or absence of Si (2 mM) (C: control plants not exposed to HM). Values represent normalized relative quantities (NRQs). For each group, the Pearson coefficient is provided. The table represents statistical analyses of the heatmap hierarchical clustering. The different letters indicate that the values are significantly different from each other (*p* < 0.05; Tukey’s HSD all-pairwise comparisons).

The hierarchical clustering of the expression profiles [represented as a heatmap; Figures 3(leaves), 4 (roots)] for various treatments was performed using a Euclidean distance matrix in a complete linkage. The clustering resulted in a separation between control plants (C and CSi), Cd-exposed plants (Cd and CdSi), and Zn-exposed plants (Zn and ZnSi).

**FIGURE 4:**
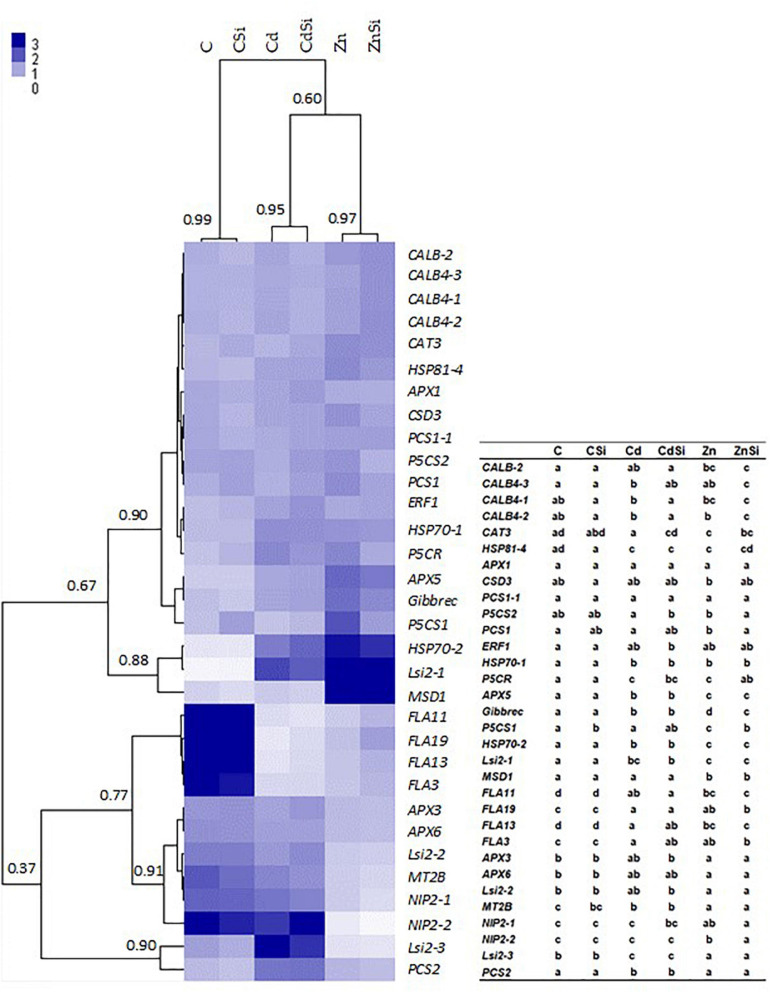
Heatmap hierarchical clustering showing the expression of genes assessed by RT-qPCR in the roots of hemp plants. Plants were exposed for 1 week to Cd (20 μM) or Zn (100 μM) in the presence or absence of Si (2 mM) (C: control plants not exposed to HM). The values represent NRQs. For each group, the Pearson’s coefficient is provided. The table represents statistical analyses of the heatmap hierarchical clustering. The different letters indicate that the values are significantly different from each other (*p* < 0.05; Tukey’s HSD all-pairwise comparisons).

In the leaves of HM-stressed plants, several transcripts were more abundant as compared to controls, notably *RCA*, *RBCS*, and *CLH1* (genes involved in photosynthesis), *CAD* (involved in lignin synthesis), *P5CR* (involved in proline biosynthesis), *ERF1* (involved in signal transduction), *FSD1* (involved in cell rescue), *PAL* (involved in phenylpropanoid pathway), and *Lsi2-1* (involved in Si accumulation). In addition, Cd exposure increased the expression of *PCS1-1* (involved in PC synthesis) and decreased the expression of *CLH2* and *Lsi2-2* compared to control plants. A higher expression of *MSD1*, *APX1*, *APX3*, *APX5*, *MT2* (the genes involved in cell rescue), *and CLH2* and a decreased expression of *PCS2* and *Lsi2-3* were observed in Zn-treated plants compared to control plants. A significant effect of Si application on the expression of genes of HM-stressed plants was detected: Si exposure increased the expression of a gene involved in cell rescue (*APX6*) and decreased the expression of *Lsi2-3* in Cd-treated plants while the abundances of transcripts coding for RCA, RBCS, ERF1, APX1, APX3, APX5, and MSD1 were lower in ZnSi-treated plants than in those exposed to Zn in the absence of Si. In control plants, Si application stimulated the expression of genes involved in photosynthesis (*FBPase* and *RCA*) and cell rescue (*CAT3, APX3, APX5, APX6, APXT, CSD3, FSD1*, and *MSD1*) while the abundance of *Lsi2-1* transcript decreased.

In roots, HM exposure increased the expression of HSPs (*HSP81-4, HSP70-1*, and *HSP70-2*), *P5CR* (proline biosynthetic gene), *APX5*, *Gibbrec*, *Lsi2-1* (Si transport), and decreased the expression of *FLAs* (*FLA3, FLA11, FLA13*, and *FLA19;* CW-related) and *MT2B*. In addition, Cd exposure also increased the expression of *CALB4-3* (intracellular signaling), *Lsi2-3*, and *PCS2* compared to control plants. A pronounced expression of *CALB-2*, *PCS1*, *MSD1* and a decreased expression of *APX3, APX6*, *Lsi2-2*, *Lsi2-3*, *NIP2-1* (Si channel), and *NIP2-2* were observed in plants when exposed to Zn. In control plants, Si exposure significantly stimulated the expression of a gene involved in proline biosynthesis (*P5CS1*). In plants exposed to Cd, Si decreased the expression of *CABL4-1* and *CABL4-2* and increased those of *CAT3* and *P5CS2*. Genes involved in intracellular signaling (*CABL4-3* and *CABL4-2*) were more expressed, and *P5CS2*, *PCS1*, *P5CR*, *Gibbrec*, *P5CS1*, and *NIP2-2* were less expressed in ZnSi-treated plants than in Zn-exposed ones.

### Proteomics

**FIGURE 5:**
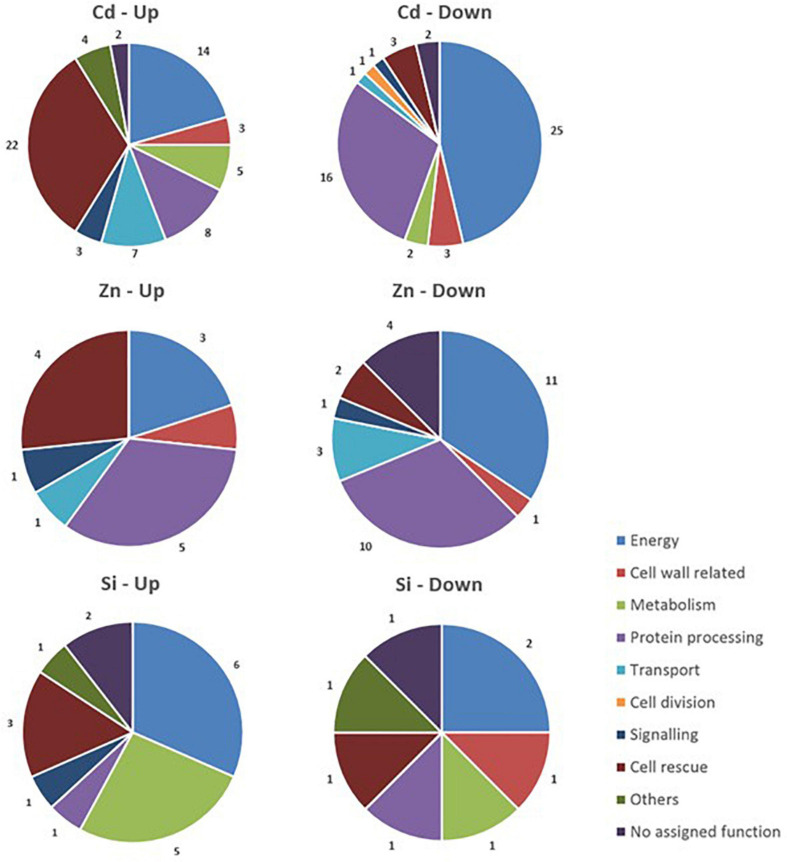
Functional classification of proteins with significant quantitative changes in abundance in hemp leaves in response to Cd, Zn, and Si. Seedlings were exposed for 1 week either to Cd 20 μM, Zn 100 μM, or Si 2 mM, and proteins were extracted from leaves. Mixed treatments (CSi, CdSi, and ZnSi) are not indicated for the sake of clarity.

The data relative to the impact of the treatments on protein regulation is provided in Table 1, which simultaneously considers soluble and membrane-bound protein fractions. The accumulation of 122 proteins was affected by Cd, whereas 47 were affected by Zn (Figure 5). Only a minor proportion of proteins (16) was affected by both Cd and Zn, suggesting a different impact of these HM on plant metabolism. The number of proteins affected by Si (27) exposure was by far lower than the number of proteins affected by HM. Detailed results are presented below.

**TABLE 1:** List of proteins with significant quantitative changes of hemp leaves in response to Cd. Zn and Si.

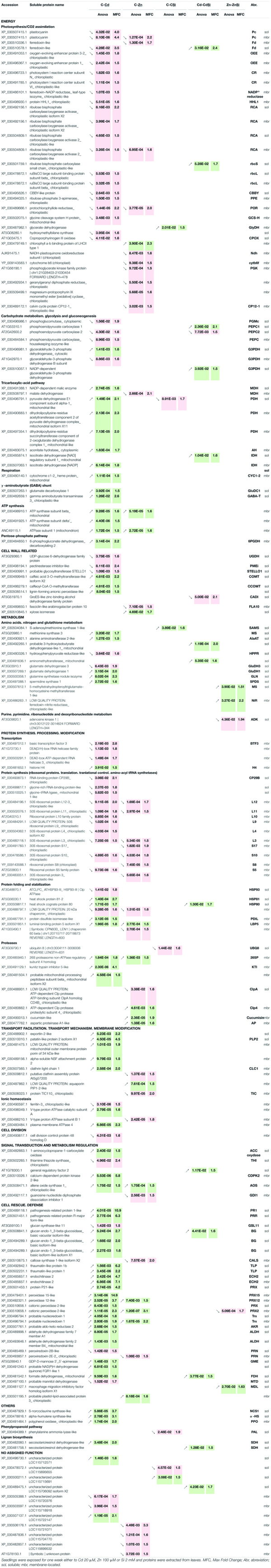

### Photosynthesis/CO_2_ Assimilation

Most of the identified proteins involved in light-dependent and light-independent reactions of photosynthesis as well as chlorophyll biosynthesis-related proteins showed reduced abundance under HM exposure.

Hemp response to Cd resulted in a significant decrease in the abundance of oxygen-evolving enhancer protein (OEE), plastocyanin (Pc), photosystem (PS) I reaction center (subunits N, VI), ferredoxin (Fd), and Fd-NADP^+^ reductase (NADP^+^ reductase), RCA, RBCS large subunit-binding protein (rbcL), CBBY, and ribulose-phosphate 3-epimerase (PPE), protochlorophyllide reductase (POR), hydroxymethylbilane synthase, and coproporphyrinogen III oxidase (CPOX). Only two proteins (Fd and rbcS) were significantly upregulated in CdSi-exposed plants in comparison to Cd-treated ones.

Under Zn exposure, NADH-plastoquinone oxidoreductase subunit I (Ndh), cytochrome b6f (cytb6f), Pc, Fd, RCA, phosphoglycerate kinase (PGK), Calvin cycle protein CP12-1 (CP12-1), POR, geranylgeranyl diphosphate reductase, and magnesium-protoporphyrin IX monomethyl ester cyclase were found to be less abundant in comparison to controls. Photosynthesis-related proteins were not affected by Si in ZnSi-exposed plants in comparison to Zn-treated ones.

In the absence of HM stress, Si exposure was shown to increase the abundance of glycerate dehydrogenase (GlyDH), a protein involved in photorespiration.

### Carbohydrate Metabolism and TCA Cycle

Proteins involved in carbohydrate metabolism and TCA cycle undergo the greatest change in accumulation under the conditions of Cd stress: a phosphoglucomutase (PGM), two phosphoenolpyruvate carboxylase (PEPC) compounds, and a glyceraldehyde-3-phosphate dehydrogenase (G3PDH) had a lower abundance, whereas an NADP-dependent malic enzyme (MDH), three components of pyruvate dehydrogenase (PDH) complex, an aconitate hydratase (AH), and an isocitrate dehydrogenase (IDH) were more abundant in comparison to unstressed plants. The application of 2 mM H_2_SiO_3_ under Cd exposure decreased the abundance of a PEPC (PEPC2), whereas the levels of PEPC1, a G3PDH, and an IDH increased comparatively to Cd treatment. Except for G3PDH, the proteins involved in carbohydrate metabolism were reduced by Cd, whereas the enzymes involved in the TCA pathway increased.

Zinc exposure decreased the abundance of an MDH. The addition of Si to Zn-treated plants had no impact on the abundance of proteins involved in carbohydrate metabolism.

In the absence of an HM stress, Si application decreased the abundance of a PDH compared to control plants.

### γ-Aminobutyrate Shunt and ATP Synthesis

Two enzymes, involved in γ-aminobutyrate (GABA) shunt, were induced under Cd exposure: glutamate decarboxylase (GluDC), which catalyzes the conversion of glutamate to GABA and GABA transaminase (GABA-T), which is responsible for GABA conversion to succinic semialdehyde.

Adenosine triphosphate synthesis is induced under HM stress as observed for Cd- and Zn-treated plants compared to the control ones.

In both unstressed and HM-stressed plants, no significant effect of Si application on the abundance of proteins involved in GABA shunt and ATP synthesis was detected.

### Proteins Involved in Cell Wall Formation

Heavy metals exposure had an impact on several enzymes involved in CW formation. CW components whose biosynthesis/assembly/degradation are affected by Cd exposure are pectin and hemicellulose [UDP-glucose 6-dehydrogenase (UGDH) and pectinesterase inhibitor (PME)], cellulose (glycosyltransferase STELLO1), and lignin (caffeic acid 3-*O*-methyltransferase (COMT), caffeoyl-CoA *O*-methyltransferase (CCOMT), and lignin-forming anionic peroxidase). Among them, lignin-associated enzymes were more abundant in Cd-treated plants, the others were less abundant. In the same treatment, Si exposure induced a decreased abundance of GroES-like Zn-binding alcohol dehydrogenase family protein (CAD).

Plants under Zn stress exhibited a lower abundance of fasciclin-like arabinogalactan protein 10 (FLA10) involved in SCW synthesis and a higher abundance of xylose isomerase (xylan metabolism). Si application did not affect these proteins.

### Amino Acids, Nitrogen, and GSH Metabolism

Cadmium exposure had an impact on the proteins involved in amino acids, nitrogen, and GSH metabolism. The enzymes involved in methionine metabolism [methionine synthase (MS)], alanine metabolism [alanine aminotransferase (AlaAT)], a spermidine synthase (SPDS), and a glutamine synthetase (GLN) were upregulated, two isoforms of glutamate dehydrogenase (GluDH) had altered abundance and a hydroxyphenylpyruvate reductase (HPPR) constitute a biosynthetic pathway from tyrosine to 4-hydroxyphenyllactic acid (pHPL) was downregulated. In this treatment, Si application increased the abundance of a protein involved in the pathway of L-valine degradation (probable 3-hydroxyisobutyrate dehydrogenase-like) and an aminomethyltransferase.

Zinc exposure had no impact on this part of the metabolism. However, the plants of ZnSi treatment exhibited an increased abundance of a methyltetrahydropteroyltriglutamate-homocysteine methyltransferase (MS) involved in methionine metabolism and an Fd-nitrite reductase (NiR) involved in nitrogen assimilation comparatively to plants of Zn treatment.

In the absence of an HM stress, the abundance of an *S*-adenosylmethionine synthase (SAM) was increased after Si exposure.

### Protein Synthesis, Processing, and Modification

Heavy metals exposure affected the enzymes involved in protein synthesis, processing, and modification. In both Cd and Zn treatments, the abundance of proteins involved in synthesis is significantly decreased while most of the proteases identified were more abundant. Two proteins involved in folding and stabilization showed the same variation of abundance under Cd or Zn stress: the 20 kDa chaperonin was less abundant and luminal-binding protein 5 was more abundant. Cd treatment also increased the abundance of HSPs (HSP81 and HSP80), a protein disulfide-isomerase, and decreased the abundance of HSP93. Zn-exposed plants had a lower abundance of chaperonin 60 beta. Si application only had an impact on the abundance of an HSP80 in Cd-treated plants and an oligo-ubiquitin involved in proteolysis (UBQ8) in control plants.

### Aquaporin, Transport Facilitation, Transport Mechanism, and Membrane Modification

In Cd treatment, plants exhibited higher levels of an exportin (protein export from the nucleus), a patatin-like protein 2 (PLP2, phospholipase activity), a porin, and two proteins involved in vesicular trafficking (alpha-soluble NSF attachment protein 2; clathrin light chain 1, CLC1).

Zinc exposure also affected vesicular trafficking by lowering the abundance of a putative clathrin assembly protein and a voltage-dependent cation-selective channel Translocon of the inner chloroplast membrane (TIC), and by increasing the abundance of an aquaporin (PIP1-2).

Silicon did not affect this class of proteins.

### Ionic Homeostasis

Cadmium and Zn had a different impact on the subunits of V-type proton ATPase. In Cd-treated plants, the abundance of the A subunit increased while in Zn treatment B subunit was less abundant. Cd exposure also increased the abundance of another plasma membrane ATPase and decreased the abundance of ferritin (ferritin three), involved in iron buffering.

Silicon did not affect this class of proteins.

### Signal Transduction and Metabolism Regulation

Most of the proteins with a function in signal transduction and metabolism regulation were upregulated under Cd stress: an 1-aminocyclopropane-1-carboxylate oxidase (ACC oxidase), involved in ethylene synthesis; a calcium-dependent protein kinase (CDPK), and an allene oxide synthase (AOS, involved in jasmonate synthesis) (Farmer and Goossens, 2019) were more abundant. In these plants, thiamine thiazole synthase (THI) was less abundant. In *Arabidopsis*, THI1 interacts with Ca^2+^-dependent protein kinase CPK33 and modulates the S-type anion channels and stomatal closure (Li et al., 2016). Compared to the plants of Cd treatment, the plants of CdSi treatment exhibited a higher abundance of the proteins identified as general regulatory factor two.

In Zn treatment, two proteins had a different abundance compared to the control ones: AOS was more abundant while a guanosine nucleotide diphosphate dissociation inhibitor (GDI) regulates the activity of rho of plants (ROP) proteins. Si exposure had no impact on Zn-treated plants for this class of proteins.

### Cell Rescue, Defense

Most of the cell rescue proteins induced by Cd are part of two functional classes: pathogenesis-related proteins (PR) and oxidative stress response. Cd exposure upregulated PR1, a PR-related protein (PRR), two thaumatin-like proteins (TLP), two endochitinases (ECH2), and three callose-associated enzymes (glucan endo-1,3-beta-glucosidases, BG). Oxidative stress response-related proteins with a higher abundance in Cd-treated plants were: peroxidases (PRX2, PRX12, PRX15, and protein P21-like), two probable nucleoredoxins (Trx), a probable aldo-keto reductase (AKR), two aldehyde dehydrogenases (ALDH), a probable NAD(P)H dehydrogenase (quinone) FQR1-like1, and a GDP-D-mannose 3′–5′-epimerase (GME), which regulate ascorbate synthesis under stress conditions and adjust the balance between ascorbate and CW monosaccharide biosynthesis. The abundance of formate dehydrogenase (FDH) was also increased under Cd exposure while a probable mannitol dehydrogenase (MTD), a peroxiredoxin (PRN), and a glucan synthase-like 11 (GSL11) were less abundant compared to control. In the same treatment, Si application increased the abundance of an FDH and a BG.

Zinc exposure increased not only the abundance of proteins involved in oxidative stress response (PRX2, PRX12, and Trx) but also that of a probable plastid-lipid-associated protein. Zn-treated plants also exhibited a decreased abundance of a callose synthase (CALS) and a PRN. Si application under Zn stress induced a higher abundance of a macrophage migration inhibitory factor homolog (MDL, involved in stress response pathways) and decreased the abundance of a PRX2 comparatively to Zn-treated plants in the absence of Si.

### Others

Cadmium-stressed plants exhibited a higher abundance of a secoisolariciresinol dehydrogenase-like (SDH) protein, involved in lignan biosynthesis. Another SDH was more abundant in CdSi treatments compared to Cd treatment.

In the absence of HM stress, Si had an impact on the phenylpropanoid pathway: PAL was less abundant.

### Microscopic Determination of Lignification and Cd Deposition in Leaves and Roots

**FIGURE 6:**
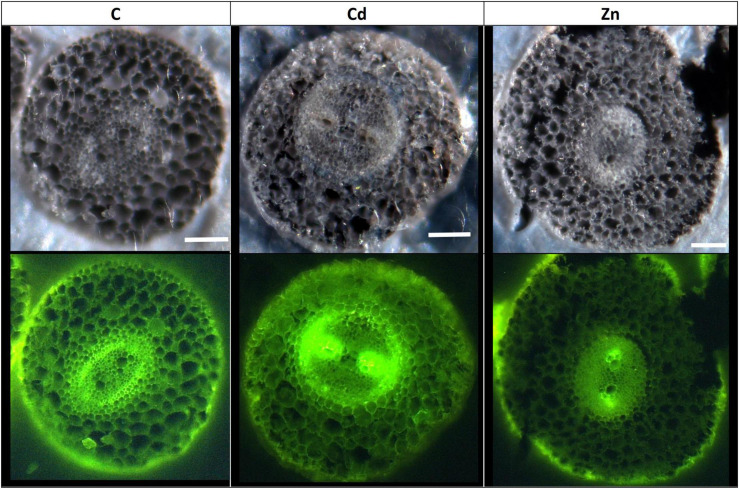
Confocal microscope observation of hemp root sections (60 μm) (Axioscope 2 MOT, 405 nm). Plants were exposed for 1 week to Cd (20 μM) or Zn (100 μM) (C: control plants not exposed to HM). Fluorescence highlights lignified areas (yellow). Scale bar: 100 μm.

Confocal microscopy images (Supplementary Figure 2) pointed out an increase of lignified areas in the leaves of Cd treatment compared to the control ones. Pictures obtained in ESRF showed that Cd is mostly localized in these lignified areas of Cd-treated plants (Supplementary Figure 3). In CdSi treatments, Cd was also detected in leaf trichomes while it was absent from trichomes in the leaves of Cd treatment. In roots, confocal microscopy images (Figure 6) showed an increased lignification of the stele in Cd treatment, and an increased lignification of the exodermis in Zn treatment compared to the control ones.

## Discussion

**FIGURE 7:**
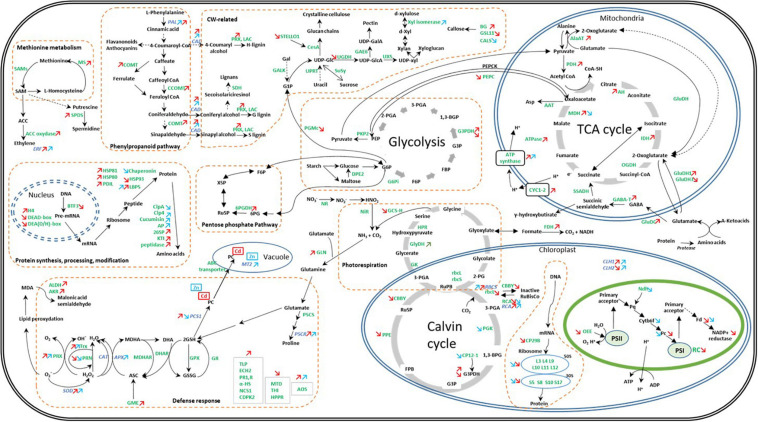
Global overview of the impact of Cd and Zn on the abundance of proteins and transcripts involved in the cell metabolism in hemp leaves. Seedlings were exposed for 1 week either to Cd 20 μM, Zn 100 μM, or Si 2 mM. Colored arrows indicate a significant effect of Zn (in blue) or Cd (in red). Adapted from Liu et al. (2018).

Zinc and Cd are frequently and simultaneously found in HM-contaminated soils (Feng et al., 2020). Those elements share numerous chemical properties and in this study induce a comparable range of growth inhibition in *C. sativa*. However, we also demonstrated that Cd and Zn clearly acted on distinct cellular targets, and this is valid for both gene expression and protein abundance (Figure 7).

### Stress Sensing and ROS Management

Stress sensing constitutes the first step of plant response to deleterious environmental conditions. Proteins/genes involved in signal perception were found to be higher in abundance upon HM exposure. These include Ca^++^ signaling-related proteins/genes in the leaves of Cd treatment (CDPK), and the roots of Cd and Zn treatments [calcium-dependent lipid-binding proteins (*CALB*)], and AOS in both HM treated plants. CALB proteins have been proposed to partake in intracellular signaling upon stress (de Silva et al., 2011), and many AOSs generate precursors of the defense hormone jasmonate (Farmer and Goossens, 2019).

Ethylene has dual functions in plants as it acts as a major determinant of plant senescence but also as a key mediator of the biotic and abiotic stress response (Müller and Munné-Bosch, 2015). SAM is the precursor of ethylene, and MS catalyzes the conversion of homocysteine to methionine, which is further converted into SAM by SAM synthetase (Hossain et al., 2012). The increased abundance of ethylene biosynthesis-related proteins (MS and ACC oxidase) has been observed in plants exposed to Cd but not in response to Zn (Figure 7). In contrast, we observed a similar increased expression of *ERF1* in the leaves of plants exposed to Cd or Zn. *ERF1* is involved in both ethylene and jasmonic acid signaling pathways (Müller and Munné-Bosch, 2015). Furthermore, Müller and Munné-Bosch (2015) suggest that *ERFs* might regulate ROS-responsive gene expression, thereby conferring stress tolerance. Several cell rescue proteins that accumulated in Cd- and Zn-exposed plants were indeed found to be oxidoreductases, indicating detoxification-mediated tolerance in the plant. Peroxidases and a probable nucleoredoxin (Trx), involved in the regulation of antioxidant enzymes (Kneeshaw et al., 2017) were more abundant in stressed plants compared to control ones, as well as the transcript abundances of the leaves of *SOD*s (FSD) and *APX*s.

Cadmium had a stronger impact compared to Zn on proteins involved in cell rescue and defense (Figure 7). However, Zn exposure had a stronger impact on *APX*, *SOD*, and *CAT* gene expression than Cd: in roots, it decreased the gene expression of *APX3* and *APX6* while *APX5, MSD1*, and *CAT3* were more expressed. In the leaves of Zn-exposed plants, *APX1*, *APX3, APX5, MSD1*, and *FSD1* were more expressed compared to controls. Cd exposure only affected the root expression of *APX5* (increased) and expression of *FSD1* in the leaves (increased). To cope with ROS-induced reactive aldehydes, plants under Cd stress also exhibited increased leaf content of AKR and ALDH (Figure 7), catalyzing reactions leading to less toxic alcohols and carboxylic acids, respectively (Sengupta et al., 2015; Vemanna et al., 2017; Ahmad et al., 2019), while this type of strategy was not involved in the response to Zn. Cd-exposed plants also increased the abundance of a PLP2 with phospholipase activity in plants exposed to Cd. The patatin-related phospholipase A (pPLA) might be involved in the removal of oxidatively modified fatty acids from membranes in membrane remodeling and repair (Yang et al., 2012).

### Cell Wall Retention and Intracellular Compartmentation

The retention of HM in the CW constitutes an efficient strategy to limit their accumulation in the symplasm, and lignification may contribute to making the CW less permeable for toxic ions (Pejic et al., 2009; Ye et al., 2012; Fernández et al., 2014; Vukcevic et al., 2014; Gutsch et al., 2019; Nawaz et al., 2019). In this study, hemp plants subjected to an excess of Zn or Cd showed an increased expression of *PAL* and *CAD* involved in lignin biosynthesis in the leaves (Figure 7). Lignin monomers are synthesized through the phenylpropanoid pathway, of which PAL catalyzes the initial step (Gutsch et al., 2019). However, as far as protein abundances are concerned, only Cd had a detectable impact on the enzymes involved in lignin synthesis (COMT; CCOMT; lignin-forming anionic peroxidase; GroES-like Zn-binding alcohol dehydrogenase family protein, CADI). These results correlate with the rise in the lignified areas observed on confocal microscopy images (Figure 6 and Supplementary Figure 2), which indicate lignification zones in Cd-treated roots and leaves but not in Zn-exposed plants.

Once inside the symplasm, the regulation of a plasmodesmata aperture through callose synthesis and deposition may limit the transfer of metal ions from one cell to another (Singh et al., 2016; O’Lexy et al., 2018). While a decrease in plasmodesmata permeability may be considered as an attempt to ion sequestration in some cells, the opposite reaction formed to increase permeability should be regarded as an attempt of dilution at the whole tissue level. Interestingly, the alterations in the abundance of callose-associated enzymes (GSL11; glucan endo-1,3-beta-glucosidase (BG); and CALS) were observed under HM exposure, and we suspected Cd and Zn to decrease callose content and increase plasmodesmata permeability by acting on two distinct targets. Glucan endo-1,3-β-D-glucosidases or β-1,3-glucanase (BG) are often referred to as PR because of their ability to hydrolyze β-1,3-glucan chains of fungal CWs (Hong et al., 2002) but the one identified in the present case is lysosomal and so not involved in the degradation of fungi CW. In this study, the isoforms corresponding to these enzymes were strongly increased in response to Cd stress but not in response to Zn. Conversely, Zn reduced CALS, thus suggesting that Zn reduces the synthesis of callose while Cd increased its degradation.

Chelating metals by forming a metal complex of PCs or metallothioneins (MTs) at the intracellular and intercellular level are part of the mechanisms used by plants to counteract HM toxicity (Alloway, 2012; Emamverdian et al., 2015). In the root level, the gene coding for MT2 was not induced in response to Cd, and it was even slightly repressed by Zn, suggesting that this metallothionein did not afford key protection in the roots of HM-treated plants. It has to be noticed, however, that *MT2* was induced at the leaf level in plants exposed to Zn both in the presence and the absence of Si. On the contrary, we detected a strong accumulation of PC in Cd-treated roots (Figure 2C), which could be related to an induction of *PCS2* expression in Cd-treated roots while *PCS1-1* expression remained unmodified comparatively to control plants. As far as leaves are concerned, a significant increase in *PCS2* was observed in Zn-treated plants, which could explain PC accumulation; however, Cd-treated plants still accumulated PC at the leaf level and for this treatment, only *PCS1-1* was overexpressed comparatively to control. The fact that PC may translocate from the root to the shoot may partially modify the relationship between PC synthase gene expression at a given organ and PC concentration recorded in this precise organ.

The complex formed between a metal ion and a chelatant is transported to the vacuoles where the ion will be released and fixed to organic acids (Cobbett, 2000; Jost and Jost-Tse, 2018). Other transporters may also directly transfer free HM to the vacuole. This study also revealed the accumulation of cation/proton exchanger in Cd-treated plants (V-type proton ATPase catalytic subunit A and plasma membrane ATPase 4), while the opposite trend was observed under Zn exposure (decreased abundance of V-type proton ATPase subunit B1). The induction of vacuolar pumps (V-ATPase and V-PPase), together with a set of tonoplast transporters and primary ATP-dependent pumps allows vacuolar compartmentalization of HM (Sharma et al., 2016). In addition to direct transport over the tonoplast membrane, vesicular transport may play a role in vacuolar sequestration or efflux from the cell (Leitenmaier and Küpper, 2011). In Cd treatment, plants exhibited higher levels of two proteins involved in vesicular trafficking: CLC1 and alpha-soluble NSF attachment protein 2. Clathrin is composed of two heavy chain subunits (CHC1 and CHC2) and two light chain subunits (CLC1 and CLC2). The subunits assemble a cage-like scaffold around developing vesicles to support vesicle formation. Once the vesicle is fully formed, this scaffolding dissociates and is detached from the initial membrane by dynamin, allowing the vesicle to traffic to its destination (reviewed by Larson et al., 2017). At the destination, vesicle fusion to membranes is mediated by soluble NSF attachment protein receptor (SNARE) complexes that assemble from subunits present at both the plasma membrane and the surface of the docking vesicles (Larson et al., 2017): NSF is associated with membranes by soluble NSF attachment protein binding (Lee et al., 2009). The stimulation of vesicular trafficking under Cd exposure may be linked to the HM sequestration or efflux from the cell, but could also reflect a strategy to regulate the internalization of critical plasma membrane proteins involved in hormone signaling, stress responses, and nutrient uptake (Wan and Zhang, 2012; Wang et al., 2013). Surprisingly, Zn had the opposite effect by lowering the abundance of a putative clathrin assembly protein.

### Photosynthetic Processes, TCA Cycle, and Carbohydrate Metabolism

A recent study demonstrated that Cd-induced a decrease in all photosynthetic pigments leading to a decrease in net photosynthesis (Luyckx et al., 2021a). Zn is also known to have a deleterious effect on leaf photosynthesis (Lefèvre et al., 2014). The present study, once again, demonstrated that Cd and Zn acted on distinct photosynthetic-related proteins as only three recorded proteins were affected by both Cd and Zn.

Cadmium reduced the light-absorbing efficiency of PSs by decreasing the abundance of enzymes involved in steps 4, 7, and 13 (hydroxymethylbilane synthase, CPOX, and POR, respectively) of chlorophyll synthesis (Beale, 2005), and by decreasing the abundance of OEE complex (the donor side of PSII), and PSI reaction center-related proteins. Zn also affected the abundance of photosynthetic pigments by decreasing the abundance of enzymes involved in steps 10 (magnesium-protoporphyrin IX monomethyl ester cyclase) and 13, but no impact on a PS reaction center was detected. Furthermore, in Zn-treated plants, the increased expression of two CLH (*CLH1* and *CLH2*) was observed while Cd had a contrasting effect on *CLH* expression. Both Cd and Zn altered the accumulation of protein involved in electron transport (Fd and Pc) with a specific impact of Zn on a Cytb6f complex and Pq abundances. It can be assumed that the stress impairment of the light reactions will decrease ATP and NADPH production, necessary for the CO_2_ reduction process. Cd and Zn also limited the abundance of proteins involved in the Calvin cycle. Cd negatively affected the abundance of a protein involved in carbon fixation (RCA and rbcL), G3PDH abundance, and ribulose 1,5-bisphosphate (RuBP) regeneration (PPE and CBBY), whereas Zn mostly affected enzymes involved in the fixation of CO_2_ (RCA and PGK) and CP12-1. In leaves, the increased expression of Calvin cycle-linked transcripts (*RCA* and *RBCS*) was observed under HM stress, probably to counteract the negative effect of HM on RBCS activity, Zn having a higher impact than Cd on *RBCS* expression.

The toxic effects of Cd and Zn can be linked to the ability of HM to bind, SH groups of pigment biosynthetic enzymes, their ability to supersede the functionality of essential metal ions and indirectly to ROS-induced oxidation and degradation of proteins (Bashir et al., 2015). HM toxicity also entails the impairment of protein biosynthesis: Cd or Zn exposure had a negative impact on transcription and protein biosynthesis, and most of the proteins detected had a chloroplastic localization (Cd: 62% and Zn: 100%). Zn exposure also decreased the abundance of a voltage-dependent cation-selective channel (TIC). The translocon at the inner envelope membrane of chloroplasts (TIC) plays a central role in plastid biogenesis by coordinating the sorting of nucleus-encoded preproteins across the inner membrane and coordinating the interactions of preproteins with the processing and folding machinery of the stroma (Inaba et al., 2005). The downregulation of TIC upon Zn stress probably reduced the accumulation of a variety of plastid proteins. Taken together, these results suggest a reduction of the overall photosynthetic capacity of hemp plants exposed to HM. When photosynthesis is not well operating, plants need to upregulate metabolic pathways such as glycolysis and TCA cycle to maintain the normal growth and development and sustain defenses strategies (Ben Ghnaya et al., 2009; Ashraf and Harris, 2013; Hossain and Komatsu, 2013; Smirnova et al., 2017). In this study, Cd-treated plants exhibited a higher abundance of TCA cycle proteins. ATP synthases were also found to be highly abundant under stress conditions. The upregulation of glycolysis and the TCA cycle might help the stressed plant to produce more reducing power to compensate for the high-energy demand of a Cd-challenged cell (Hossain and Komatsu, 2013).

Many defense strategies also depend on N metabolism. The accumulation of soluble N compounds (such as glycinebetaine, spermidine, and proline) in plants facing adverse environmental constraints may help regulate osmotic potential in cells, and protect and stabilize the membranes, thus improving HM stress tolerance (Hussain et al., 2020). This work demonstrates that proline accumulated in the leaves but not in the roots of HM-exposed plants. In addition to a role in the regulation of cell osmotic potential, proline can act as a metal chelator, an antioxidative defense molecule, and a signaling molecule during stress (Kaur and Asthir, 2015; Siddique et al., 2018). The last step of proline synthesis from pyrroline-5-carboxylate is catalyzed by P5CR, and the corresponding gene was upregulated in HM-treated plants. *P5CS1* was specifically upregulated in the roots of Zn-exposed plants, but the resulting increase in proline content remained insignificant considering the high level of variability.

Heavy metals exposure can severely hamper nitrogen metabolism by reducing NO_3_^–^ uptake (the alteration of membrane permeability) and altering the activity of various N assimilatory enzymes by binding to the vital-SH groups (Ahsan et al., 2009; Hussain et al., 2020). It is therefore crucial for plants to be able to maintain N assimilation under stress. GLN/glutamate synthase (GOGAT) is the main pathway of NH_4_^+^ assimilation into nontoxic glutamine and glutamate under normal conditions (Ahsan et al., 2009; Hussain et al., 2020). GLN plays also a role in the re-assimilation of ammonium released during photorespiration. Photorespiratory N cycling might be 10 times higher than primary N assimilation (Kaminski et al., 2015). When the endogenous NH_4_^+^ concentration increases, i.e., in response to Cd toxicity, an alternative pathway, controlled by GluDH, contributes to lowering this internal NH_4_^+^ concentration (Hussain et al., 2020). GluDH is also involved in the recycling of carbon molecules by supplying 2-oxoglutarate to tissues becoming carbon limited (Dubois et al., 2003). A higher abundance of GLN and an isoform of GluDH under Cd exposure (Figure 7) may indicate a rise in photorespiration and the ability of hemp plants to elevate nitrogen and carbon use efficiency to deal with stress conditions. Additionally, Cd was able to trigger the activation of the GABA shunt through an increased abundance of GluDC while Zn had no impact (Figure 7). The synthesis of GABA could contribute to the dissipation of excess energy and release CO_2_ allowing the Calvin cycle to function. According to Carillo (2018), GABA shunt can supply NADH to the mitochondrial transport chain under the conditions in which the TCA cycle is impaired, but in Cd-treated plants, numerous proteins are involved in the TCA cycle increased suggesting that this pathway may adequately react to Cd toxicity.

It has to be mentioned that an increase in gene expression did not necessarily lead to a recorded increase in the corresponding protein abundances. This was the case for the enzymes involved in PC synthesis (see the description above) and for the enzymes involved in proline synthesis, whose abundance did not increase. Discrepancies between transcriptomic and proteomic data have already been discussed by Kubala et al. (2015). In this study, it was demonstrated that Cd stress, and to a lesser extent Zn toxicity, decreased the abundance of a wide range of proteins regulating protein synthesis while increasing the abundance of numerous proteases, and this could add an additional level of complexity, which hamper the correlation between the gene expression and the abundance of corresponding gene products.

### Impact of Si Supply on Gene Expression and Protein Abundance in Hemp Facing or Not HM Stress

Silicon is considered beneficial to plants under stress conditions (Ma et al., 2001). In a previous study, we showed that hemp can absorb Si from nutrient solution and translocate this element to the aerial parts of the plant (Guerriero et al., 2019; Luyckx et al., 2021a,b). In this study, we demonstrate that Zn-treated plants exposed to Si accumulated higher amounts of Si in their roots than the plants exposed to Si alone. Similarly, Cd-treated plants exposed to Si exhibited a higher concentration of Si in their leaves than the plants exposed to Si alone. Moreover, Si induced Cd accumulation in leaf trichomes (Figure 7). According to Ma et al. (2015) and Kim et al. (2017), such behavior suggests that increasing Si content may be regarded as an attempt to improve stress tolerance. An increased accumulation of the well-known osmoprotectant proline in the leaves of CdSi-treated plants compared to Cd-exposed ones supports this hypothesis.

Silicon accumulation in plants is well described in rice, and ortholog genes to rice Si channels and efflux transporters have been found in hemp (Guerriero et al., 2019). In this study, *C. sativa* genes corresponding to the rice efflux transporter *OsLsi2* on one hand and *NIP2-1* and *NIP2-2* corresponding to the rice Si channel *OsLsi* mediating silicic acid passage (Guerriero et al., 2019) were analyzed. In the root level, *CsaLsi2-1* expression was increased by HM treatment. *CsaLsi2-3* was increased in Cd-treated plants but was surprisingly reduced in Zn-treated plants as it was the case for *CsaLsi2-2*. It is tempting to speculate that *CsaLsi2-1* mainly contributes to Si accumulation in the roots of Zn-treated plants, although it has to be explained how this could occur considering the *NIP2-1* and *NIP2-2* expressions were also decreased by Zn treatments. As far as leaves are concerned, *CsaLsi2-1* was also increased by HM exposure. When supplemented with Si, control plants and plants exposed to Cd or Zn globally exhibited a decreased gene expression of Si channels and efflux transporter. Plants were exposed to Si a week before HM exposure. We may assume that when a protective concentration of Si is reached in plants, increased transcription of Si channels/efflux transporters is no longer needed.

The idea of a protecting effect of Si is supported by the number of proteins (27) affected by Si exposure comparatively to the plants grown in the absence of Si. Moreover, of the 27 proteins with an altered abundance after Si exposure: 7 were modified in control plants, and 20 were found in the plants exposed to HM treatments. It is noteworthy that among these 20 proteins, 12 were not modified by HM treatment in the absence of Si, suggesting that Si does not only counteract the impact of HM but also confers a specific physiological status to stressed plants. This is supported by the fact that none of these proteins were modified by Si in the absence of HM. Moreover, Cd and Zn had a quite distinct impact on hemp proteome (see the details mentioned earlier), and it is therefore not unexpected that no common protein appeared to be modified by both CdSi and ZnSi treatments.

In control plants, Si supplementation stimulated the Calvin cycle by increasing the expression of *FBPase* and *RCA* but also increased the abundance of GlyDH, a protein involved in photorespiration. It has already been suggested that Si-enhanced stress tolerance is linked to the accumulation of photorespiratory enzymes (Frew et al., 2018). Even if plants were not exposed to HM, basic metabolic processes can impart the stress on plants (Frew et al., 2018). Supplementing plants with Si also tended to stimulate proline synthesis in leaves (*P5CS1* and *P5CS2*) and roots (*P5CR*) and plant oxidative response capacity [increased expression of *CAT*, *APX*, and *SOD* (*FSD*)]. This underlines the potential importance of Si for basic metabolic processes and not only for plant response to external stresses (Frew et al., 2018).

In plants exposed to HM, energy metabolism is of crucial importance to sustain the cost of metabolic adaptations the plant needs to set up. A beneficial effect of Si on energy metabolism was observed under Cd exposure only: light-dependent reactions (Fd), light-independent reactions (*FBPase* and *RCA* expression, rbcS abundance), glycolysis (G3PDH), and TCA cycle (IDH) were stimulated in the plants of CdSi treatment comparatively to the plants of Cd treatment in the absence of Si.

As discussed earlier, N metabolism represents another important component of defense strategies. Si supply was associated with an increased NiR (N assimilation) abundance under Zn exposure, and an increased expression of *P5CS2* in the roots of Cd-treated plants. Proline, and PC and MT are associated with metal chelation. While the expression of MT and PCS isoforms tended to increase in Cd-treated plants supplemented with Si, the opposite trend occurred in the plants of ZnSi treatment compared to the plants of Zn treatment. It has already been suggested that the retention of HM in the CW is probably the first strategy in response to metal entry in plants and that lignin-bound silica in CWs improves the metal binding and reduces the metal ion transfer (Ye et al., 2012; Fernández et al., 2014; Nawaz et al., 2019). Once inside the cells, NPTs and organic acids might also participate in HM tolerance (Fernández et al., 2014). We can assume that Si-mediated compartmentation is sufficient to lower excess ion concentration of Zn and do not require another sequestration strategy given that, unlike Cd, Zn is an essential element for plants and is also shown to be preferentially bound to O/N ligands rather than to PC (Lefèvre et al., 2014). HM exposure is usually followed by ROS accumulation. Si application was shown to improve the oxidative response capacity in the leaves and roots of plants exposed to Cd (*APX6* and *CAT3*) but decreased the root expression of APXs (*APX1,3,5*) and SOD (*MSD1*), and PRX2 abundance in the leaves of plants exposed to Zn.

Silicon had an impact on signal transduction: supplementing plants with Si induced a decreased root expression of CALBs in the plants of Cd treatment and *Gibbrec* in the plants of Zn treatment but increased the expression of CALBs in the roots of Zn treatment. Surprisingly, Si decreased the expression of *ERF1* in the leaves of Zn-treated plants.

## Conclusion

Zinc and Cd are frequently and simultaneously found in HM-contaminated soils. HM exposure had a negative impact on photosynthesis and protein synthesis and induced a comparable range of reactions in *C. sativa:* the stimulation of antioxidants, the chelation and compartmentation of metal ions, and the upregulation of glycolysis and TCA cycle. However, we demonstrated that Cd and Zn acted on distinct cellular targets and this is valid for both gene expression and protein abundance.

To devise agricultural strategies aimed at improving crop yield, the effect of Si on the stress tolerance of plants was considered. In this study, we demonstrate that HM-treated plants exposed to Si accumulated higher amounts of Si than controls. Si was shown to counteract the impact of HM but also to confer a specific physiological status to stressed plants, with a quite distinct impact on the hemp proteome of CdSi and ZnSi treatments.

## Data Availability Statement

The original contributions presented in the study are included in the article/Supplementary Material, further inquiries can be directed to the corresponding author.

## Author Contributions

SL, J-FH, and GG coordinated the project. ML, SL, and GG conceived and designed the experiment. ML performed all the experimental analyses. GG contributed to the transcriptomic data analysis. KS contributed to the proteomic data analysis. ML and SL wrote the manuscript. All authors reviewed and approved the final manuscript.

## Conflict of Interest

The authors declare that the research was conducted in the absence of any commercial or financial relationships that could be construed as a potential conflict of interest.

## Publisher’s Note

All claims expressed in this article are solely those of the authors and do not necessarily represent those of their affiliated organizations, or those of the publisher, the editors and the reviewers. Any product that may be evaluated in this article, or claim that may be made by its manufacturer, is not guaranteed or endorsed by the publisher.

## Supplementary Material

Supplementary Figure 1 Dry weight (g) of the roots, stems, and leaves of *Cannabis sativa* (cv. Santhica 27). Plants were exposed for 1 week to cadmium (Cd) (20 μM) or zinc (Zn) (100 μM) in the presence or absence of silicon (Si) (2 mM) [C: control plants not exposed to heavy metals (HM)]. The different letters indicate that the values are significantly different from each other (*p* < 0.05; Tukey’s HSD all-pairwise comparisons).

Supplementary Figure 2 Confocal microscope observation of hemp leaf sections (60 μm) (Axioscope 2 MOT, 405 nm). Plants were exposed for 1 week to Cd (20 μM) or Zn (100 μM) (C: control plants not exposed to HM). Fluorescence highlights lignified areas (yellow), and areas containing chlorophyll (orange-red color). Scale bar: 200 μm.

Supplementary Figure 3 Synchrotron-ID21. Cd and Si distribution in the hemp leaf sections (60 μm) of Cd- and CdSi-exposed plants. Plants were exposed for 1 week to Cd (20 μM) (C: control plants not exposed to HM). Red arrows: trichomes.
