# Supplementary figures and images for: Molecular and Biochemical Insights Into Early Responses of Hemp to Cd and Zn Exposure and the Potential Effect of Si on Stress Response
Source: Front Plant Sci. 2021 Sep 3;12:711853. doi: 10.3389/fpls.2021.711853 (PMC8446647; doi:10.3389/fpls.2021.711853)

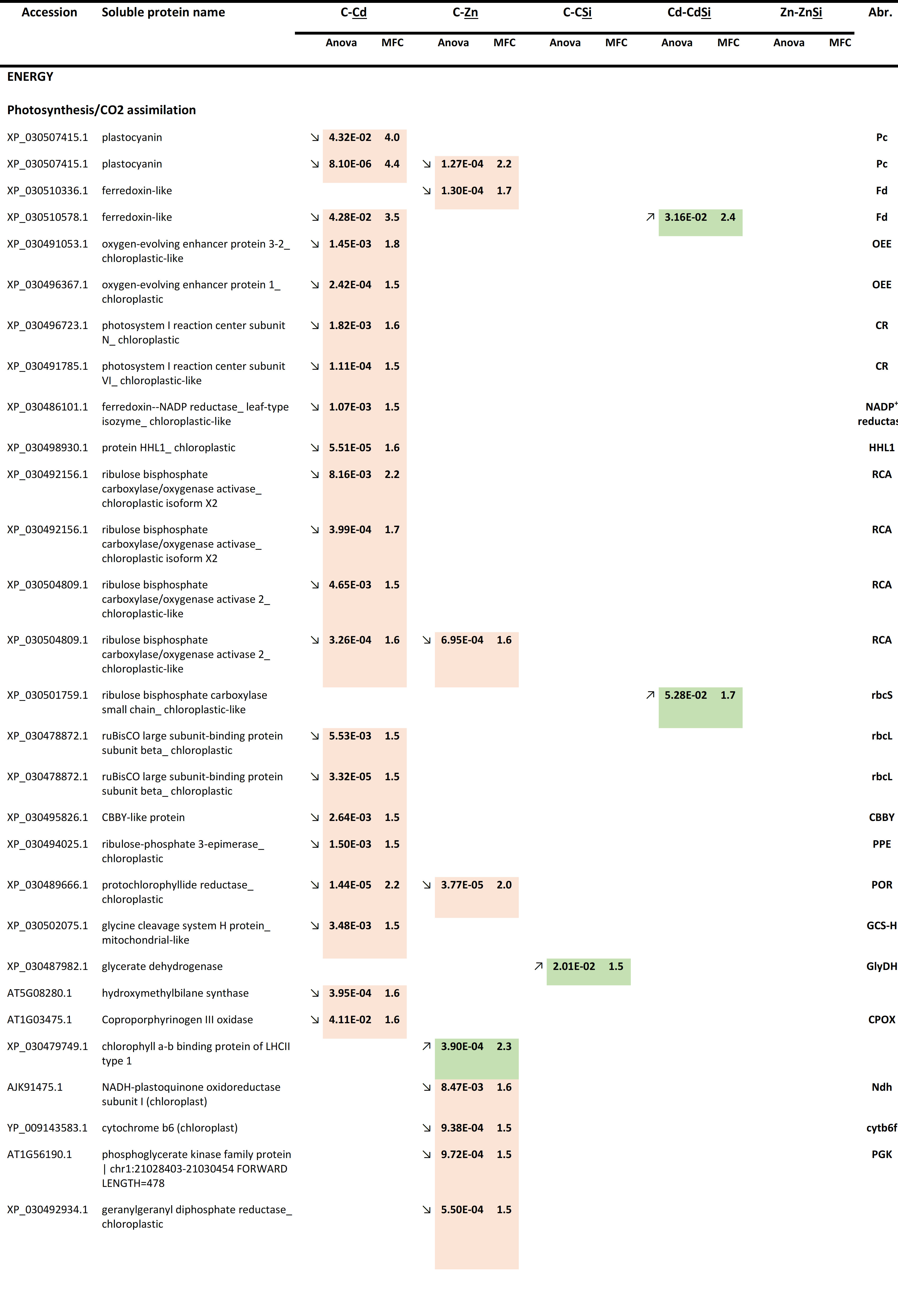

Supplement: Supplementary Figure 1 — Dry weight (g) of the roots, stems, and leaves of Cannabis sativa (cv. Santhica 27). Plants were exposed for 1 week to cadmium (Cd) (20 μM) or zinc (Zn) (100 μM) in the presence or absence of silicon (Si) (2 mM) [C: control plants not exposed to heavy metals (HM)]. The different letters indicate that the values are significantly different from each other (p < 0.05; Tukey’s HSD all-pairwise comparisons). [file Image_1.png]

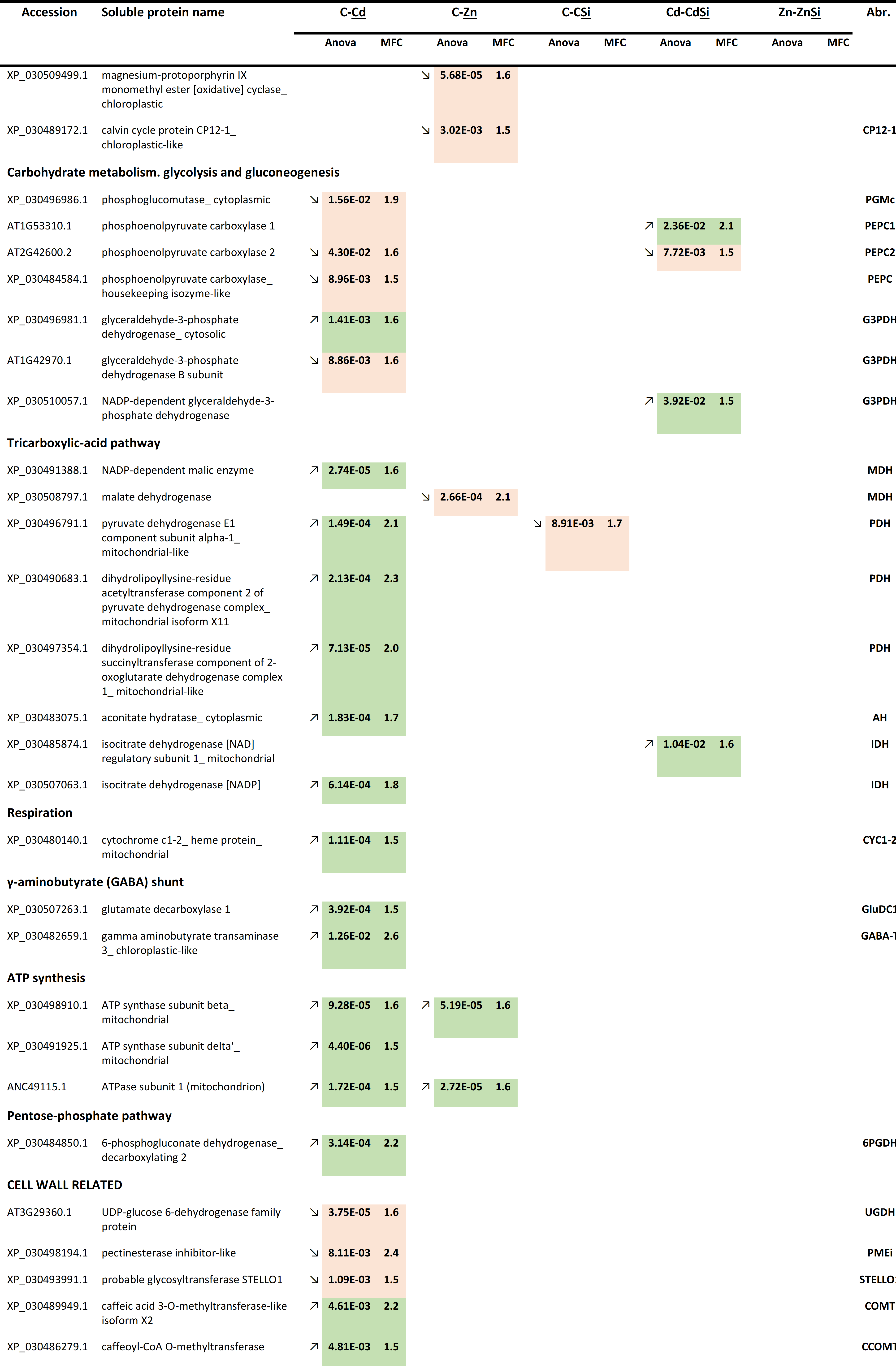

Supplement: Supplementary Figure 2 — Confocal microscope observation of hemp leaf sections (60 μm) (Axioscope 2 MOT, 405 nm). Plants were exposed for 1 week to Cd (20 μM) or Zn (100 μM) (C: control plants not exposed to HM). Fluorescence highlights lignified areas (yellow), and areas containing chlorophyll (orange-red color). Scale bar: 200 μm. [file Image_2.png]

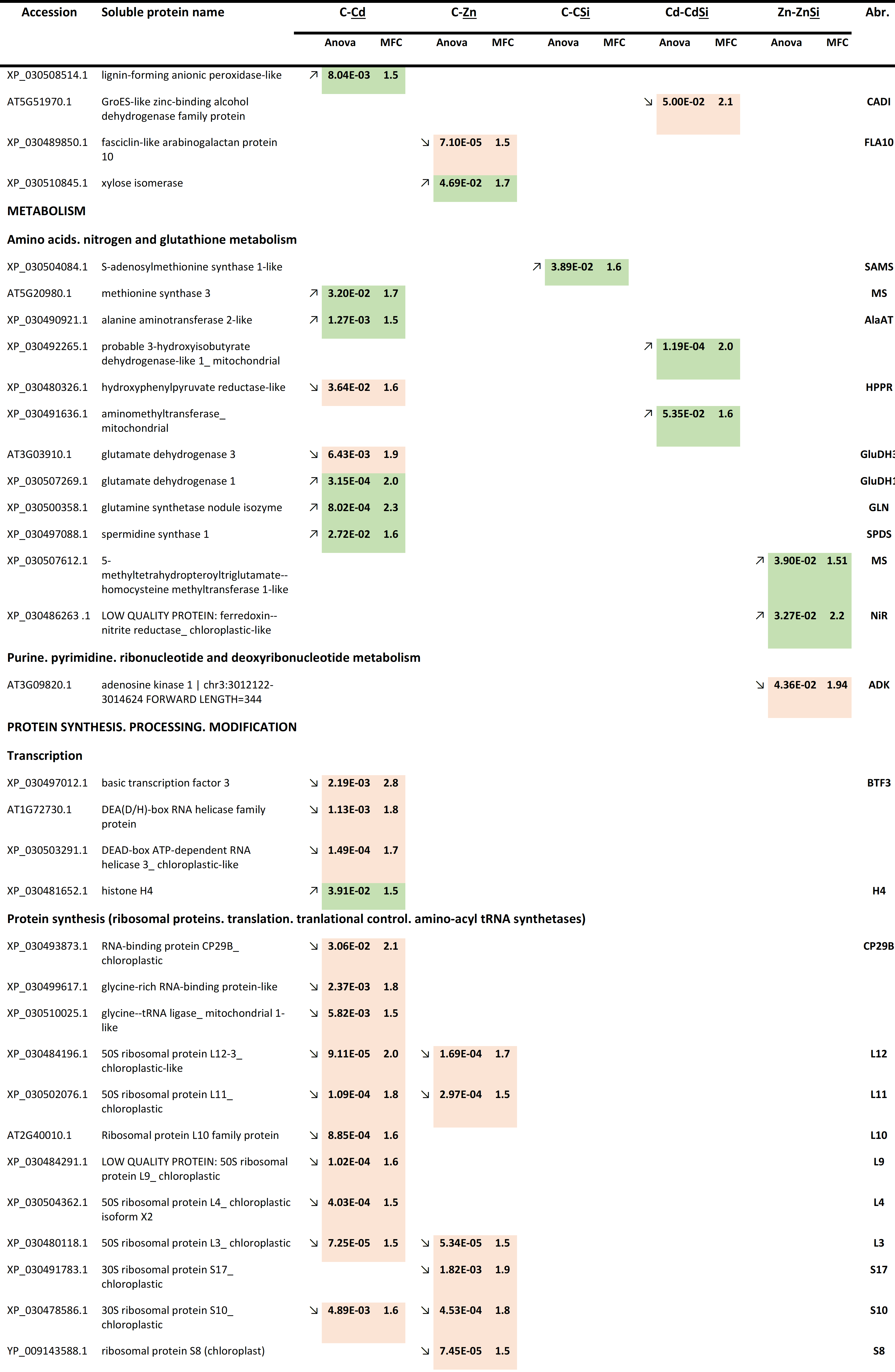

Supplement: Supplementary Figure 3 — Synchrotron-ID21. Cd and Si distribution in the hemp leaf sections (60 μm) of Cd- and CdSi-exposed plants. Plants were exposed for 1 week to Cd (20 μM) (C: control plants not exposed to HM). Red arrows: trichomes. [file Image_3.png]

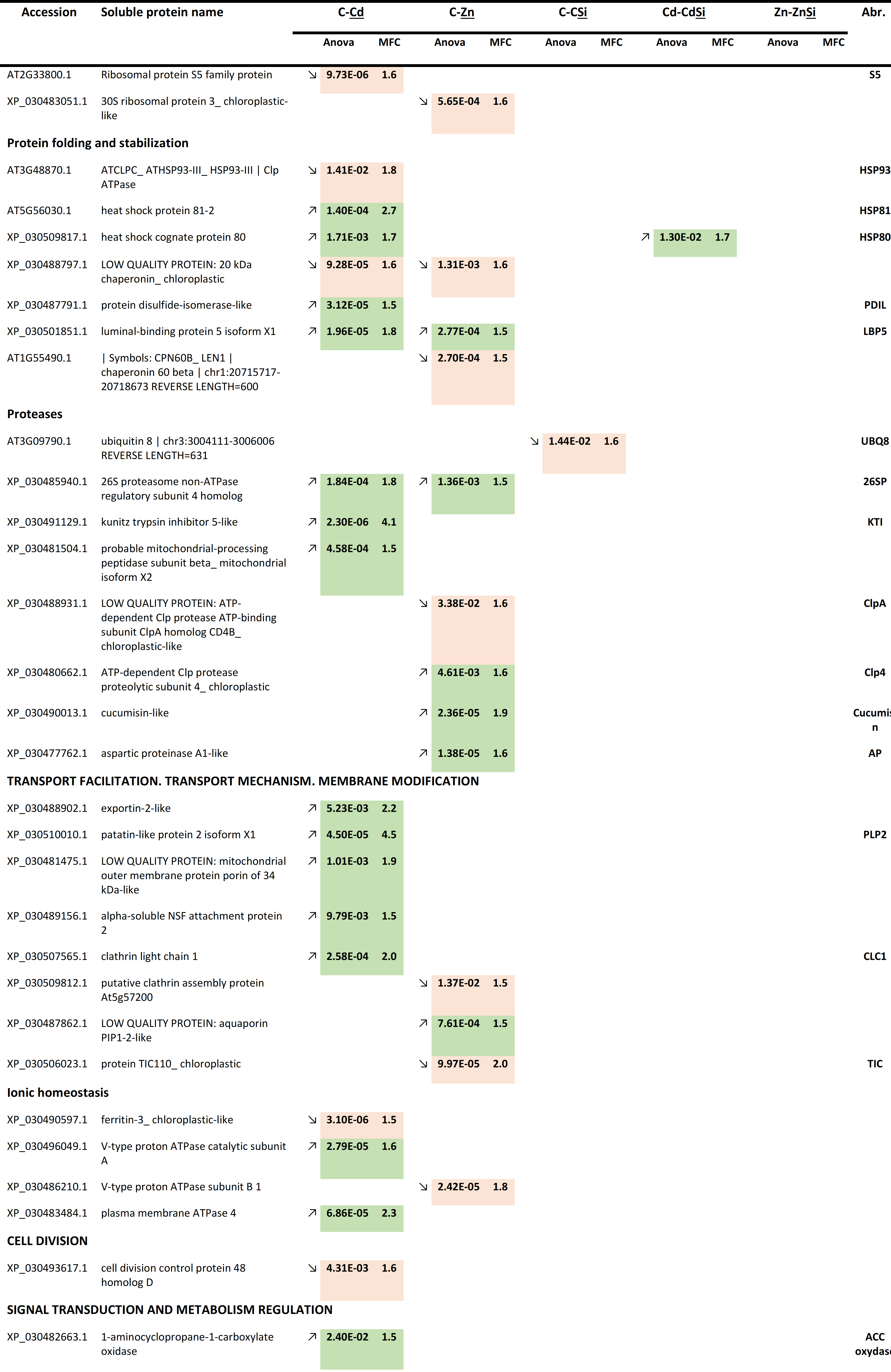

Supplement: Supplementary file 4 [file Image_4.png]

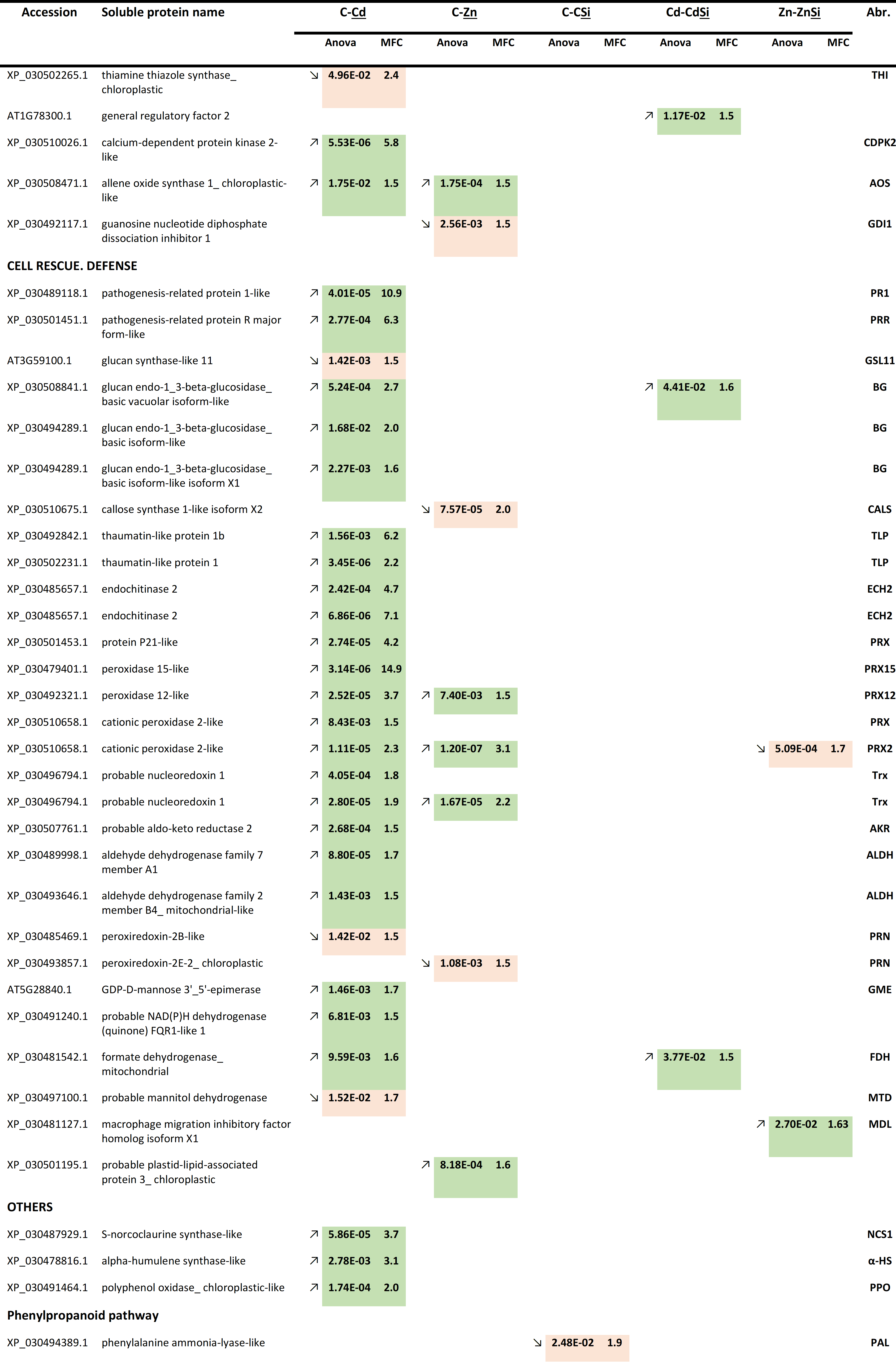

Supplement: Supplementary file 5 [file Image_5.png]

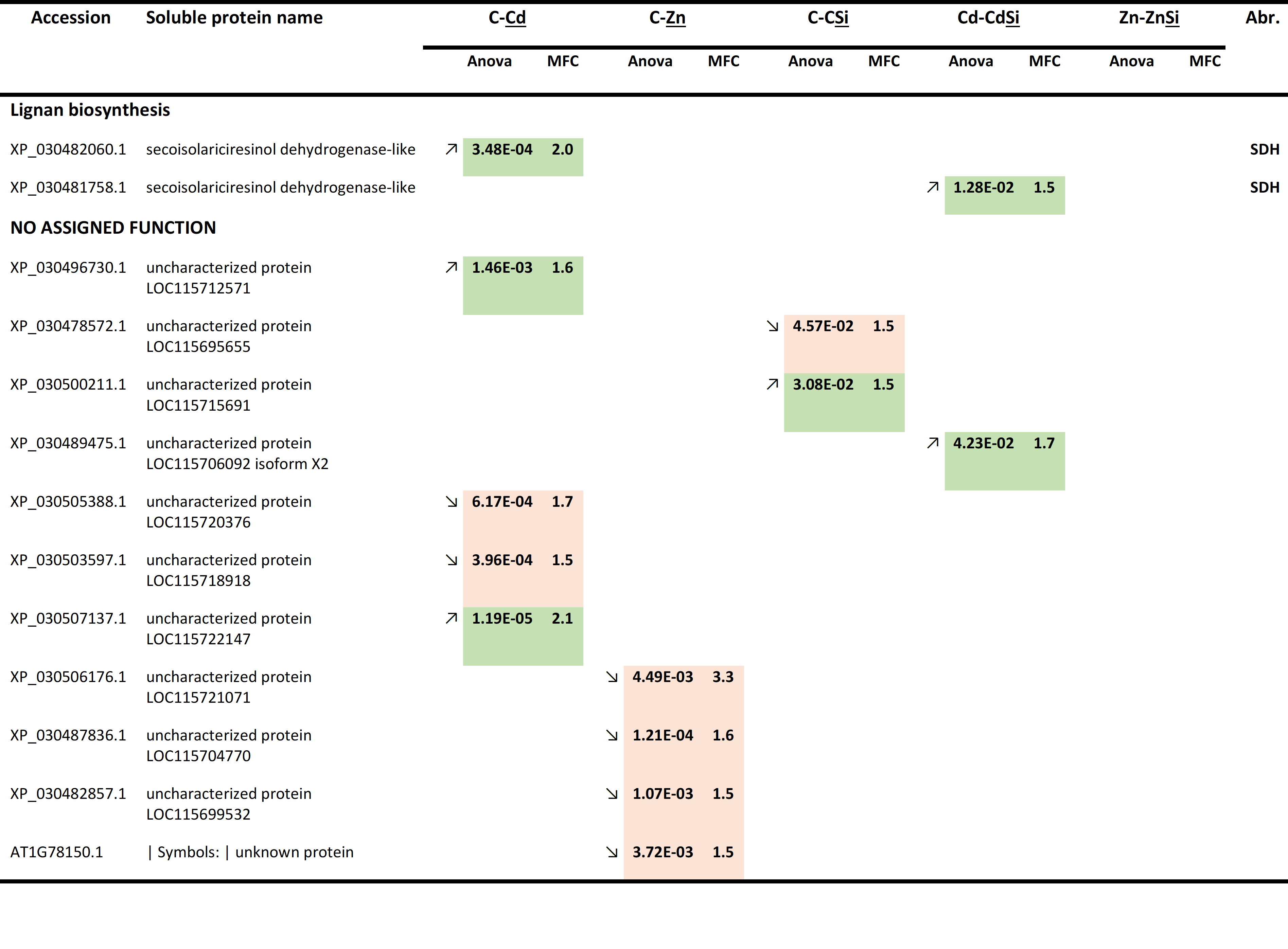

Supplement: Supplementary file 6 [file Image_6.png]
