# Supplementary material for: Molecular and Biochemical Insights Into Early Responses of Hemp to Cd and Zn Exposure and the Potential Effect of Si on Stress Response
Source: Front Plant Sci. 2021 Sep 3;12:711853. doi: 10.3389/fpls.2021.711853 (PMC8446647; doi:10.3389/fpls.2021.711853)
Supplement: Supplementary file 7 [file Table_1.pdf]

Table1: List of proteins with significant quantitative changes of hemp leaves in response to Cd. Zn and Si. Seedlings were exposed for one week either to Cd 20 µM, Zn 100 µM or Si 2 mM and proteins were extracted from leaves. MFC: Max Fold Change. Abr.: abreviation; sol: soluble; mbr: membrane-located.

Accession	Soluble protein name	C-Cd		C-Zn		C-CSi		Cd-CdSi		Zn-ZnSi		Abr.	
		Anova	MFC	Anova	MFC	Anova	MFC	Anova	MFC	Anova	MFC		
**ENERGY**													
**Photosynthesis/CO2 assimilation**													
XP_030507415.1	plastocyanin	↘ 4.32E-02	4.0									Pc	sol
XP_030507415.1	plastocyanin	↘ 8.10E-06	4.4	↘ 1.27E-04	2.2							Pc	mbr
XP_030510336.1	ferredoxin-like			↘ 1.30E-04	1.7							Fd	mbr
XP_030510578.1	ferredoxin-like	↘ 4.28E-02	3.5					↗ 3.16E-02	2.4			Fd	sol
XP_030491053.1	oxygen-evolving enhancer protein 3-2_ chloroplastic-like	↘ 1.45E-03	1.8									OEE	mbr
XP_030496367.1	oxygen-evolving enhancer protein 1_ chloroplastic	↘ 2.42E-04	1.5									OEE	mbr
XP_030496723.1	photosystem I reaction center subunit N_ chloroplastic	↘ 1.82E-03	1.6									CR	mbr
XP_030491785.1	photosystem I reaction center subunit VI_ chloroplastic-like	↘ 1.11E-04	1.5									CR	mbr
XP_030486101.1	ferredoxin--NADP reductase_ leaf-type isozyme_ chloroplastic-like	↘ 1.07E-03	1.5									$NADP^+$ reductase	mbr
XP_030498930.1	protein HHL1_ chloroplastic	↘ 5.51E-05	1.6									HHL1	mbr
XP_030492156.1	ribulose bisphosphate carboxylase/oxygenase activase_ chloroplastic isoform X2	↘ 8.16E-03	2.2									RCA	sol
XP_030492156.1	ribulose bisphosphate carboxylase/oxygenase activase_ chloroplastic isoform X2	↘ 3.99E-04	1.7									RCA	mbr
XP_030504809.1	ribulose bisphosphate carboxylase/oxygenase activase 2_ chloroplastic-like	↘ 4.65E-03	1.5									RCA	sol
XP_030504809.1	ribulose bisphosphate carboxylase/oxygenase activase 2_ chloroplastic-like	↘ 3.26E-04	1.6	↘ 6.95E-04	1.6							RCA	mbr
XP_030501759.1	ribulose bisphosphate carboxylase small chain_ chloroplastic-like							↗ 5.28E-02	1.7			rbcS	sol
XP_030478872.1	ruBisCO large subunit-binding protein subunit beta_ chloroplastic	↘ 5.53E-03	1.5									rbcL	sol
XP_030478872.1	ruBisCO large subunit-binding protein subunit beta_ chloroplastic	↘ 3.32E-05	1.5									rbcL	mbr
XP_030495826.1	CBBY-like protein	↘ 2.64E-03	1.5									CBBY	sol
XP_030494025.1	ribulose-phosphate 3-epimerase_ chloroplastic	↘ 1.50E-03	1.5									PPE	mbr
XP_030489666.1	protochlorophyllide reductase_ chloroplastic	↘ 1.44E-05	2.2	↘ 3.77E-05	2.0							POR	mbr
XP_030502075.1	glycine cleavage system H protein_ mitochondrial-like	↘ 3.48E-03	1.5									GCS-H	mbr
XP_030487982.1	glycerate dehydrogenase					↗ 2.01E-02	1.5					GlyDH	mbr
AT5G08280.1	hydroxymethylbilane synthase	↘ 3.95E-04	1.6										sol
AT1G03475.1	Coproporphyrinogen III oxidase	↘ 4.11E-02	1.6									CPOX	sol
XP_030479749.1	chlorophyll a-b binding protein of LHCII type 1			↗ 3.90E-04	2.3								mbr
AJK91475.1	NADH-plastoquinone oxidoreductase subunit I (chloroplast)			↘ 8.47E-03	1.6							Ndh	mbr
YP_009143583.1	cytochrome b6 (chloroplast)			↘ 9.38E-04	1.5							cytb6f	mbr
AT1G56190.1	phosphoglycerate kinase family protein | chr1:21028403-21030454 FORWARD LENGTH=478			↘ 9.72E-04	1.5							PGK	mbr
XP_030492934.1	geranylgeranyl diphosphate reductase_ chloroplastic			↘ 5.50E-04	1.5								mbr

Accession	Soluble protein name	C-Cd		C-Zn		C-CSi		Cd-CdSi		Zn-ZnSi		Abr.	
		Anova	MFC	Anova	MFC	Anova	MFC	Anova	MFC	Anova	MFC		
XP_030509499.1	magnesium-protoporphyrin IX monomethyl ester [oxidative] cyclase_ chloroplastic			↘ 5.68E-05	1.6								mbr
XP_030489172.1	calvin cycle protein CP12-1_ chloroplastic-like			↘ 3.02E-03	1.5							CP12-1	mbr
**Carbohydrate metabolism. glycolysis and gluconeogenesis**													
XP_030496986.1	phosphoglucomutase_ cytoplasmic	↘ 1.56E-02	1.9									PGMc	sol
AT1G53310.1	phosphoenolpyruvate carboxylase 1							↗ 2.36E-02	2.1			PEPC1	sol
AT2G42600.2	phosphoenolpyruvate carboxylase 2	↘ 4.30E-02	1.6					↘ 7.72E-03	1.5			PEPC2	sol
XP_030484584.1	phosphoenolpyruvate carboxylase_ housekeeping isozyme-like	↘ 8.96E-03	1.5									PEPC	mbr
XP_030496981.1	glyceraldehyde-3-phosphate dehydrogenase_ cytosolic	↗ 1.41E-03	1.6									G3PDH	sol
AT1G42970.1	glyceraldehyde-3-phosphate dehydrogenase B subunit	↘ 8.86E-03	1.6									G3PDH	mbr
XP_030510057.1	NADP-dependent glyceraldehyde-3-phosphate dehydrogenase							↗ 3.92E-02	1.5			G3PDH	sol
**Tricarboxylic-acid pathway**													
XP_030491388.1	NADP-dependent malic enzyme	↗ 2.74E-05	1.6									MDH	sol
XP_030508797.1	malate dehydrogenase			↘ 2.66E-04	2.1							MDH	mbr
XP_030496791.1	pyruvate dehydrogenase E1 component subunit alpha-1_ mitochondrial-like	↗ 1.49E-04	2.1			↘ 8.91E-03	1.7					PDH	sol
XP_030490683.1	dihydrolipoyllysine-residue acetyltransferase component 2 of pyruvate dehydrogenase complex_ mitochondrial isoform X11	↗ 2.13E-04	2.3									PDH	mbr
XP_030497354.1	dihydrolipoyllysine-residue succinyltransferase component of 2-oxoglutarate dehydrogenase complex 1_ mitochondrial-like	↗ 7.13E-05	2.0									PDH	mbr
XP_030483075.1	aconitate hydratase_ cytoplasmic	↗ 1.83E-04	1.7									AH	mbr
XP_030485874.1	isocitrate dehydrogenase [NAD] regulatory subunit 1_ mitochondrial							↗ 1.04E-02	1.6			IDH	sol
XP_030507063.1	isocitrate dehydrogenase [NADP]	↗ 6.14E-04	1.8									IDH	mbr
**Respiration**													**sol**
XP_030480140.1	cytochrome c1-2_ heme protein_ mitochondrial	↗ 1.11E-04	1.5									CYC1-2	mbr
**γ-aminobutyrate (GABA) shunt**													
XP_030507263.1	glutamate decarboxylase 1	↗ 3.92E-04	1.5									GluDC1	sol
XP_030482659.1	gamma aminobutyrate transaminase 3_ chloroplastic-like	↗ 1.26E-02	2.6									GABA-T	sol
**ATP synthesis**													
XP_030498910.1	ATP synthase subunit beta_ mitochondrial	↗ 9.28E-05	1.6	↗ 5.19E-05	1.6								mbr
XP_030491925.1	ATP synthase subunit delta'_ mitochondrial	↗ 4.40E-06	1.5										mbr
ANC49115.1	ATPase subunit 1 (mitochondrion)	↗ 1.72E-04	1.5	↗ 2.72E-05	1.6								mbr
**Pentose-phosphate pathway**													
XP_030484850.1	6-phosphogluconate dehydrogenase_ decarboxylating 2	↗ 3.14E-04	2.2									6PGDH	mbr
**CELL WALL RELATED**													
AT3G29360.1	UDP-glucose 6-dehydrogenase family protein	↘ 3.75E-05	1.6									UGDH	sol
XP_030498194.1	pectinesterase inhibitor-like	↘ 8.11E-03	2.4									PMEi	sol
XP_030493991.1	probable glycosyltransferase STELLO1	↘ 1.09E-03	1.5									STELLO1	sol
XP_030489949.1	caffeic acid 3-O-methyltransferase-like isoform X2	↗ 4.61E-03	2.2									COMT	sol
XP_030486279.1	caffeoyl-CoA O-methyltransferase	↗ 4.81E-03	1.5									CCOMT	sol

Accession	Soluble protein name	C-Cd		C-Zn		C-CSi		Cd-CdSi		Zn-ZnSi		Abr.	
		Anova	MFC	Anova	MFC	Anova	MFC	Anova	MFC	Anova	MFC		
XP_030508514.1	lignin-forming anionic peroxidase-like	↗ 8.04E-03	1.5										sol
AT5G51970.1	GroES-like zinc-binding alcohol dehydrogenase family protein							↘ 5.00E-02	2.1			CADI	sol
XP_030489850.1	fasciclin-like arabinogalactan protein 10			↘ 7.10E-05	1.5							FLA10	mbr
XP_030510845.1	xylose isomerase			↗ 4.69E-02	1.7								sol
**METABOLISM**													
**Amino acids. nitrogen and glutathione metabolism**													
XP_030504084.1	S-adenosylmethionine synthase 1-like					↗ 3.89E-02	1.6					SAMS	sol
AT5G20980.1	methionine synthase 3	↗ 3.20E-02	1.7									MS	sol
XP_030490921.1	alanine aminotransferase 2-like	↗ 1.27E-03	1.5									AlaAT	sol
XP_030492265.1	probable 3-hydroxyisobutyrate dehydrogenase-like 1_ mitochondrial							↗ 1.19E-04	2.0				sol
XP_030480326.1	hydroxyphenylpyruvate reductase-like	↘ 3.64E-02	1.6									HPPR	sol
XP_030491636.1	aminomethyltransferase_ mitochondrial							↗ 5.35E-02	1.6				sol
AT3G03910.1	glutamate dehydrogenase 3	↘ 6.43E-03	1.9									GluDH3	sol
XP_030507269.1	glutamate dehydrogenase 1	↗ 3.15E-04	2.0									GluDH1	sol
XP_030500358.1	glutamine synthetase nodule isozyme	↗ 8.02E-04	2.3									GLN	sol
XP_030497088.1	spermidine synthase 1	↗ 2.72E-02	1.6									SPDS	sol
XP_030507612.1	5-methyltetrahydropteroyltriglutamate--homocysteine methyltransferase 1-like									↗ 3.90E-02	1.51	MS	sol
XP_030486263 .1	LOW QUALITY PROTEIN: ferredoxin--nitrite reductase_ chloroplastic-like									↗ 3.27E-02	2.2	NiR	mbr
**Purine. pyrimidine. ribonucleotide and deoxyribonucleotide metabolism**													
AT3G09820.1	adenosine kinase 1 | chr3:3012122-3014624 FORWARD LENGTH=344									↘ 4.36E-02	1.94	ADK	sol
**PROTEIN SYNTHESIS. PROCESSING. MODIFICATION**													
**Transcription**													
XP_030497012.1	basic transcription factor 3	↘ 2.19E-03	2.8									BTF3	mbr
AT1G72730.1	DEA(D/H)-box RNA helicase family protein	↘ 1.13E-03	1.8										mbr
XP_030503291.1	DEAD-box ATP-dependent RNA helicase 3_ chloroplastic-like	↘ 1.49E-04	1.7										mbr
XP_030481652.1	histone H4	↗ 3.91E-02	1.5									H4	mbr
**Protein synthesis (ribosomal proteins. translation. tranlational control. amino-acyl tRNA synthetases)**													
XP_030493873.1	RNA-binding protein CP29B_ chloroplastic	↘ 3.06E-02	2.1									CP29B	sol
XP_030499617.1	glycine-rich RNA-binding protein-like	↘ 2.37E-03	1.8										sol
XP_030510025.1	glycine--tRNA ligase_ mitochondrial 1-like	↘ 5.82E-03	1.5										sol
XP_030484196.1	50S ribosomal protein L12-3_ chloroplastic-like	↘ 9.11E-05	2.0	↘ 1.69E-04	1.7							L12	mbr
XP_030502076.1	50S ribosomal protein L11_ chloroplastic	↘ 1.09E-04	1.8	↘ 2.97E-04	1.5							L11	mbr
AT2G40010.1	Ribosomal protein L10 family protein	↘ 8.85E-04	1.6									L10	mbr
XP_030484291.1	LOW QUALITY PROTEIN: 50S ribosomal protein L9_ chloroplastic	↘ 1.02E-04	1.6									L9	mbr
XP_030504362.1	50S ribosomal protein L4_ chloroplastic isoform X2	↘ 4.03E-04	1.5									L4	mbr
XP_030480118.1	50S ribosomal protein L3_ chloroplastic	↘ 7.25E-05	1.5	↘ 5.34E-05	1.5							L3	mbr
XP_030491783.1	30S ribosomal protein S17_ chloroplastic			↘ 1.82E-03	1.9							S17	mbr
XP_030478586.1	30S ribosomal protein S10_ chloroplastic	↘ 4.89E-03	1.6	↘ 4.53E-04	1.8							S10	mbr
YP_009143588.1	ribosomal protein S8 (chloroplast)			↘ 7.45E-05	1.5							S8	mbr

Accession	Soluble protein name	C-Cd Anova	C-Cd MFC	C-Zn Anova	C-Zn MFC	C-CSi Anova	C-CSi MFC	Cd-CdSi Anova	Cd-CdSi MFC	Zn-ZnSi Anova	Zn-ZnSi MFC	Abr.	
AT2G33800.1	Ribosomal protein S5 family protein	↘ 9.73E-06	1.6									**S5**	mbr
XP_030483051.1	30S ribosomal protein 3_ chloroplastic-like			↘ 5.65E-04	1.6								mbr
**Protein folding and stabilization**													
AT3G48870.1	ATCLPC_ ATHSP93-III_ HSP93-III | Clp ATPase	↘ 1.41E-02	1.8									**HSP93**	sol
AT5G56030.1	heat shock protein 81-2	↗ 1.40E-04	2.7									**HSP81**	sol
XP_030509817.1	heat shock cognate protein 80	↗ 1.71E-03	1.7					↗ 1.30E-02	1.7			**HSP80**	sol
XP_030488797.1	LOW QUALITY PROTEIN: 20 kDa chaperonin_ chloroplastic	↘ 9.28E-05	1.6	↘ 1.31E-03	1.6								mbr
XP_030487791.1	protein disulfide-isomerase-like	↗ 3.12E-05	1.5									**PDIL**	mbr
XP_030501851.1	luminal-binding protein 5 isoform X1	↗ 1.96E-05	1.8	↗ 2.77E-04	1.5							**LBP5**	mbr
AT1G55490.1	| Symbols: CPN60B_ LEN1 | chaperonin 60 beta | chr1:20715717-20718673 REVERSE LENGTH=600			↘ 2.70E-04	1.5								mbr
**Proteases**													
AT3G09790.1	ubiquitin 8 | chr3:3004111-3006006 REVERSE LENGTH=631					↘ 1.44E-02	1.6					**UBQ8**	sol
XP_030485940.1	26S proteasome non-ATPase regulatory subunit 4 homolog	↗ 1.84E-04	1.8	↗ 1.36E-03	1.5							**26SP**	mbr
XP_030491129.1	kunitz trypsin inhibitor 5-like	↗ 2.30E-06	4.1									**KTI**	mbr
XP_030481504.1	probable mitochondrial-processing peptidase subunit beta_ mitochondrial isoform X2	↗ 4.58E-04	1.5										mbr
XP_030488931.1	LOW QUALITY PROTEIN: ATP-dependent Clp protease ATP-binding subunit ClpA homolog CD4B_ chloroplastic-like			↘ 3.38E-02	1.6							**ClpA**	sol
XP_030480662.1	ATP-dependent Clp protease proteolytic subunit 4_ chloroplastic			↗ 4.61E-03	1.6							**Clp4**	mbr
XP_030490013.1	cucumisin-like			↗ 2.36E-05	1.9							**Cucumisin**	mbr
XP_030477762.1	aspartic proteinase A1-like			↗ 1.38E-05	1.6							**AP**	mbr
**TRANSPORT FACILITATION. TRANSPORT MECHANISM. MEMBRANE MODIFICATION**													
XP_030488902.1	exportin-2-like	↗ 5.23E-03	2.2										sol
XP_030510010.1	patatin-like protein 2 isoform X1	↗ 4.50E-05	4.5									**PLP2**	sol
XP_030481475.1	LOW QUALITY PROTEIN: mitochondrial outer membrane protein porin of 34 kDa-like	↗ 1.01E-03	1.9										mbr
XP_030489156.1	alpha-soluble NSF attachment protein 2	↗ 9.79E-03	1.5										mbr
XP_030507565.1	clathrin light chain 1	↗ 2.58E-04	2.0									**CLC1**	mbr
XP_030509812.1	putative clathrin assembly protein At5g57200			↘ 1.37E-02	1.5								mbr
XP_030487862.1	LOW QUALITY PROTEIN: aquaporin PIP1-2-like			↗ 7.61E-04	1.5								mbr
XP_030506023.1	protein TIC110_ chloroplastic			↘ 9.97E-05	2.0							**TIC**	mbr
**Ionic homeostasis**													
XP_030490597.1	ferritin-3_ chloroplastic-like	↘ 3.10E-06	1.5										mbr
XP_030496049.1	V-type proton ATPase catalytic subunit A	↗ 2.79E-05	1.6										mbr
XP_030486210.1	V-type proton ATPase subunit B 1			↘ 2.42E-05	1.8								mbr
XP_030483484.1	plasma membrane ATPase 4	↗ 6.86E-05	2.3										mbr
**CELL DIVISION**													
XP_030493617.1	cell division control protein 48 homolog D	↘ 4.31E-03	1.6										mbr
**SIGNAL TRANSDUCTION AND METABOLISM REGULATION**													
XP_030482663.1	1-aminocyclopropane-1-carboxylate oxidase	↗ 2.40E-02	1.5									**ACC oxydase**	sol

Accession	Soluble protein name	C-Cd		C-Zn		C-CSi		Cd-CdSi		Zn-ZnSi		Abr.	
		Anova	MFC	Anova	MFC	Anova	MFC	Anova	MFC	Anova	MFC		
XP_030502265.1	thiamine thiazole synthase_ chloroplastic	↘ 4.96E-02	2.4									THI	sol
AT1G78300.1	general regulatory factor 2							↗ 1.17E-02	1.5				sol
XP_030510026.1	calcium-dependent protein kinase 2-like	↗ 5.53E-06	5.8									CDPK2	mbr
XP_030508471.1	allene oxide synthase 1_ chloroplastic-like	↗ 1.75E-02	1.5	↗ 1.75E-04	1.5							AOS	mbr
XP_030492117.1	guanosine nucleotide diphosphate dissociation inhibitor 1			↘ 2.56E-03	1.5							GDI1	mbr
**CELL RESCUE. DEFENSE**													
XP_030489118.1	pathogenesis-related protein 1-like	↗ 4.01E-05	10.9									PR1	sol
XP_030501451.1	pathogenesis-related protein R major form-like	↗ 2.77E-04	6.3									PRR	sol
AT3G59100.1	glucan synthase-like 11	↘ 1.42E-03	1.5									GSL11	sol
XP_030508841.1	glucan endo-1_3-beta-glucosidase_ basic vacuolar isoform-like	↗ 5.24E-04	2.7					↗ 4.41E-02	1.6			BG	sol
XP_030494289.1	glucan endo-1_3-beta-glucosidase_ basic isoform-like	↗ 1.68E-02	2.0									BG	sol
XP_030494289.1	glucan endo-1_3-beta-glucosidase_ basic isoform-like isoform X1	↗ 2.27E-03	1.6									BG	sol
XP_030510675.1	callose synthase 1-like isoform X2			↘ 7.57E-05	2.0							CALS	mbr
XP_030492842.1	thaumatin-like protein 1b	↗ 1.56E-03	6.2									TLP	sol
XP_030502231.1	thaumatin-like protein 1	↗ 3.45E-06	2.2									TLP	sol
XP_030485657.1	endochitinase 2	↗ 2.42E-04	4.7									ECH2	sol
XP_030485657.1	endochitinase 2	↗ 6.86E-06	7.1									ECH2	mbr
XP_030501453.1	protein P21-like	↗ 2.74E-05	4.2									PRX	sol
XP_030479401.1	peroxidase 15-like	↗ 3.14E-06	14.9									PRX15	mbr
XP_030492321.1	peroxidase 12-like	↗ 2.52E-05	3.7	↗ 7.40E-03	1.5							PRX12	mbr
XP_030510658.1	cationic peroxidase 2-like	↗ 8.43E-03	1.5									PRX	sol
XP_030510658.1	cationic peroxidase 2-like	↗ 1.11E-05	2.3	↗ 1.20E-07	3.1					↘ 5.09E-04	1.7	PRX2	mbr
XP_030496794.1	probable nucleoredoxin 1	↗ 4.05E-04	1.8									Trx	sol
XP_030496794.1	probable nucleoredoxin 1	↗ 2.80E-05	1.9	↗ 1.67E-05	2.2							Trx	mbr
XP_030507761.1	probable aldo-keto reductase 2	↗ 2.68E-04	1.5									AKR	sol
XP_030489998.1	aldehyde dehydrogenase family 7 member A1	↗ 8.80E-05	1.7									ALDH	sol
XP_030493646.1	aldehyde dehydrogenase family 2 member B4_ mitochondrial-like	↗ 1.43E-03	1.5									ALDH	sol
XP_030485469.1	peroxiredoxin-2B-like	↘ 1.42E-02	1.5									PRN	sol
XP_030493857.1	peroxiredoxin-2E-2_ chloroplastic			↘ 1.08E-03	1.5							PRN	mbr
AT5G28840.1	GDP-D-mannose 3'_5'-epimerase	↗ 1.46E-03	1.7									GME	sol
XP_030491240.1	probable NAD(P)H dehydrogenase (quinone) FQR1-like 1	↗ 6.81E-03	1.5										sol
XP_030481542.1	formate dehydrogenase_ mitochondrial	↗ 9.59E-03	1.6					↗ 3.77E-02	1.5			FDH	sol
XP_030497100.1	probable mannitol dehydrogenase	↘ 1.52E-02	1.7									MTD	sol
XP_030481127.1	macrophage migration inhibitory factor homolog isoform X1									↗ 2.70E-02	1.63	MDL	sol
XP_030501195.1	probable plastid-lipid-associated protein 3_ chloroplastic			↗ 8.18E-04	1.6								mbr
**OTHERS**													
XP_030487929.1	S-norcoclaurine synthase-like	↗ 5.86E-05	3.7									NCS1	sol
XP_030478816.1	alpha-humulene synthase-like	↗ 2.78E-03	3.1									α-HS	sol
XP_030491464.1	polyphenol oxidase_ chloroplastic-like	↗ 1.74E-04	2.0									PPO	mbr
**Phenylpropanoid pathway**													
XP_030494389.1	phenylalanine ammonia-lyase-like					↘ 2.48E-02	1.9					PAL	sol

Accession	Soluble protein name	C-Cd		C-Zn		C-CSi		Cd-CdSi		Zn-ZnSi		Abr.	
		Anova	MFC	Anova	MFC	Anova	MFC	Anova	MFC	Anova	MFC		
**Lignan biosynthesis**													
XP_030482060.1	secoisolariciresinol dehydrogenase-like	↗ **3.48E-04**	**2.0**									**SDH**	sol
XP_030481758.1	secoisolariciresinol dehydrogenase-like							↗ **1.28E-02**	**1.5**			**SDH**	sol
**NO ASSIGNED FUNCTION**													
XP_030496730.1	uncharacterized protein LOC115712571	↗ **1.46E-03**	**1.6**										sol
XP_030478572.1	uncharacterized protein LOC115695655					↘ **4.57E-02**	**1.5**						sol
XP_030500211.1	uncharacterized protein LOC115715691					↗ **3.08E-02**	**1.5**						sol
XP_030489475.1	uncharacterized protein LOC115706092 isoform X2							↗ **4.23E-02**	**1.7**				sol
XP_030505388.1	uncharacterized protein LOC115720376	↘ **6.17E-04**	**1.7**										mbr
XP_030503597.1	uncharacterized protein LOC115718918	↘ **3.96E-04**	**1.5**										mbr
XP_030507137.1	uncharacterized protein LOC115722147	↗ **1.19E-05**	**2.1**										mbr
XP_030506176.1	uncharacterized protein LOC115721071			↘ **4.49E-03**	**3.3**								mbr
XP_030487836.1	uncharacterized protein LOC115704770			↘ **1.21E-04**	**1.6**								mbr
XP_030482857.1	uncharacterized protein LOC115699532			↘ **1.07E-03**	**1.5**								mbr
AT1G78150.1	| Symbols: | unknown protein			↘ **3.72E-03**	**1.5**								mbr
